# Characterization of the Titanium(III) Tris(alkyl) Ti{CH(SiMe_3_)_2_}_3_ and its Conversion to a Dimeric Alkyl‐Bridged Titanium(IV) Species

**DOI:** 10.1002/chem.202404696

**Published:** 2025-01-28

**Authors:** Connor P. McLoughlin, Anthony J. Witt, Anni Maijala, Angela A. Shiau, Guodong Rao, R. David Britt, Heikki M. Tuononen, Philip P. Power

**Affiliations:** ^1^ Department of Chemistry University of California One Shields Avenue Davis CA 95616 United States; ^2^ Department of Chemistry NanoScience Centre University of Jyväskylä P.O. Box 35 FI-40014 Jyväskylä Finland

**Keywords:** Titanium alkyl complex, Radical decomposition, EPR spectroscopy, X-ray crystallography, Pyramidal geometry

## Abstract

The reaction of three equivalents of LiCH(SiMe_3_)_2_ with TiCl_3_(NMe_3_)_2_ afforded the rare homoleptic Ti(III) alkyl Ti{CH(SiMe_3_)_2_}_3_ (**1**) which crystallized as blue needles in 32 % yield. Single crystal X‐Ray data for **1** showed a trigonal pyramidal coordination geometry around titanium, which could be ascribed to weak interactions between the C−H bonds and the Ti(III) atom based on computational results. X‐band EPR spectroscopy gives spectral parameters consistent with the proposed Ti(III) formulation. Solutions of **1** are unstable at room temperature owing to intramolecular C−H activation that gave a dimeric Ti(IV) complex [{(Me_3_Si)_2_HC}Ti{*μ*‐CHSiMe_2_CHSiMe_3_}]_2_ (**2**).

## Graphical abstract

The trigonal pyramidal titanium(III) trialkyl is synthesized in moderate, reproducible yield via the reaction of three equivalents of Li{CH(SiMe_3_)_2_} with TiCl_3_(NMe_3_)_2_ in hexanes. The use of the latter solvent is a key feature for the success of the synthetic route. Ti{CH(SiMe_3_)_2_}_3_ is characterized by X‐ray crystallography, EPR and electronic spectroscopy.

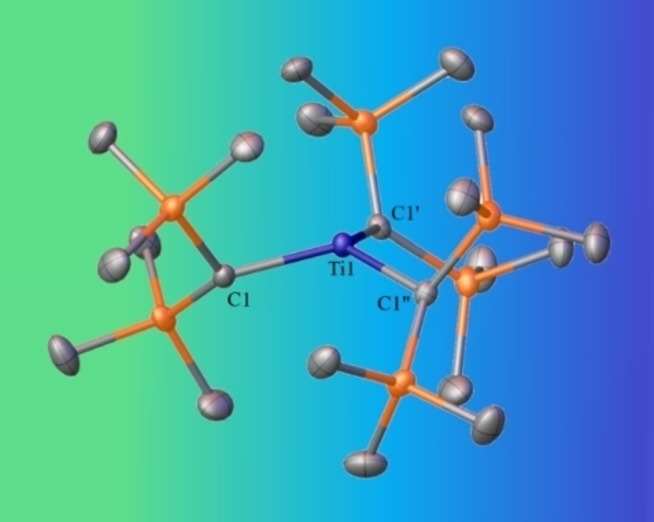

Open‐shell (d^1^‐d^9^), sigma‐bonded, homoleptic transition metal alkyls are a class of organometallic compounds that are of fundamental interest. However, their development has often been hindered by their perceived instability and the assumption that their M−C bond strengths are low.[^1^, ^2^, ^3^] In 1976, a review of metal‐sigma hydrocarbyls by Davidson, Lappert and Pearce assessed these compounds mainly on the basis of their stability.^[2]^ The latter term referred to their thermal robustness in an anhydrous, anaerobic, or inert atmosphere. The survey for the then known transition metal alkyls revealed examples for most of the metals in a range of oxidation states. However, a closer inspection of the known species revealed numerous surprising absences. The paucity of data for some species may be illustrated, at least in part, by a consideration of transition metal trialkyls. We were surprised to find that just a single example of such a species, namely the Cr(III) complex Cr{CH(SiMe_3_)_2_}_3_,^[4]^ had been structurally characterized, its residual factor was rather high; the iron centered anion FeMe_3_^−^ is also known and was observed in two crystal structures.^[5]^ Furthermore, no spectroscopic or other characterization data for Cr{CH(SiMe_3_)_2_}_3_were given. Likewise, titanium and vanadium analogues have also been reported but were stated to be extremely sensitive to handling, such that it was impossible to obtain pure samples for analytic or spectroscopic data acquisition.^[4]^ These compounds provided a sharp contrast to the corresponding isoelectronic amides M{N(SiMe_3_)_2_}_3_ (M=Sc−Co) which are all thermally stable.[^6^, ^7^, ^8^, ^9^, ^10^] We wished to synthesize and characterize stable examples of the metal trialkyls in useable yields, including a titanium derivative which has potential importance for the Ziegler‐Natta catalysis mechanism.[^11^, ^12^]

Here we describe a new synthesis and characterization of the homoleptic Ti(III) alkyl Ti{CH(SiMe_3_)_2_}_3_ (**1**).^[4]^ After extended investigation, we found that performing the synthesis in hexane instead of diethyl ether gave a blue rather than dark green solution, which, upon workup and crystallizations from diethyl ether, afforded blue‐green needles of **1** in 32 % crystalline yield – a substantial increase over the originally reported 6 %.^[4]^ The crystals were removed from a flask kept below *ca*. −20 °C. Maintaining the temperature of the sample below this threshold prolonged its stability and permitted the characterization of the highly sensitive crystals (see SI) by X‐ray crystallography (Figure 1, see SI for details).

**Figure 1:**
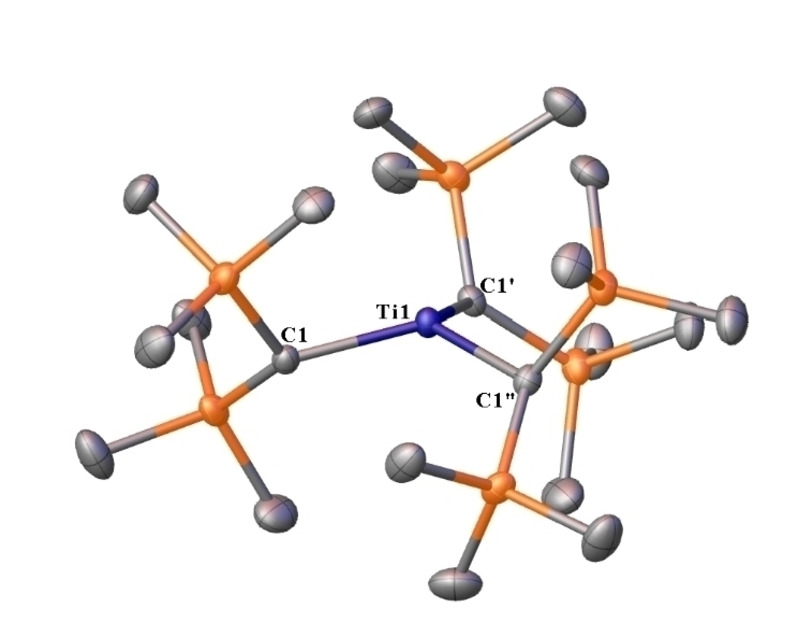
Thermal ellipsoid plot (50 %) of Ti{CH(SiMe_3_)_2_}_3_ (**1**) with hydrogen atoms not shown for clarity. Selected distances (Å) and angles (°) with calculated values in square brackets: Ti1‐C1 2.0944(17) [2.085], C1⋅⋅⋅C1′ 3.572(4) [3.543], C1‐Ti1‐C1′ 117.04(3) [116.4], C1‐C1′‐C1′′‐Ti1 −19.45(5) [−21.4].

The crystals of **1** belong to trigonal space group *P*31c and contain only one CH(SiMe_3_)_2_ ligand in the asymmetric unit. Consequently, all three Ti−C distances and C−Ti−C angles are equal. The sum of bond angles around the metal atom in **1** is 351.12(5)°, resulting in trigonal pyramidal coordination geometry. The Ti atom lies *ca*. 0.365 Å above the C1⋅⋅⋅C1′⋅⋅⋅C1“ centroid, which results in slightly greater pyramidalization for **1** in comparison to that observed for Cr{CH(SiMe_3_)_2_}_3_ which has a metal‐centroid distance of 0.324 Å.^[2]^ Interestingly, the related three‐coordinate bis(trimethylsilyl)amido complexes M{N(SiMe_3_)_2_}_3_ (M=Ti, Cr) have planar metal coordination,[^6^, ^7^, ^8^, ^9^, ^10^] which was proposed to originate from weak π‐donation from the nitrogen lone pairs to the metal or silyl atoms.^[4]^ It has also been suggested that the pyramidalization in Cr{CH(SiMe_3_)_2_}_3_ minimizes interligand contacts and provides favorable orientation of C−H moieties.^[4]^

However, neither assumption has been substantiated and no further rationale has been presented to date. Consequently, the electronic structure of **1** was modelled using density functional theory (PBE1PBE‐GD3‐BJ/def2‐TZVP, see SI for details) and subjected to bonding analyses.

The optimized structure of **1** well reproduces the key structural features of its solid‐state geometry (Figure 1). The presence of numerous interligand H⋅⋅⋅H contacts shorter than the sum of van der Waals radii for two hydrogen atoms (*ca*. 2.5 Å) suggests that instead of minimizing repulsion, the pyramidal structure of **1** could be maximizing intramolecular dispersion. However, a re‐optimization of the structure of **1** without empirical dispersion correction resulted in only minimal changes to metrical parameters. An analysis of the torsional bending potential of **1** revealed an energy requirement of only 11 kJ mol^−1^ for planarization, indicating that very subtle interactions can cause the observed pyramidal geometry. Consequently, attention was directed to the propeller‐type arrangement of bis(trimethylsilyl)methyl ligands in **1**, which allows the methine hydrogen atoms in one ligand to interact with atoms in an adjacent one. Natural Bond Orbital (NBO) analysis showed that many donor‐acceptor stabilizations are present, the most significant of which include donation from C_α_−Si_β_, Si_β_−C_γ_, or C_α_−H bonds to the lone vacant or Rydberg orbitals at the metal. However, the interactions involving the C_α_−H bonds are of primary importance to pyramidalization as substitution of the methine hydrogen atoms with methyl groups led to a trigonal planar coordination geometry around the Ti(III) center. In a similar fashion, the optimized geometry of TiMe_3_ is strongly pyramidal, whereas that of Ti(SiH_3_)_3_ is planar. Thus, the pyramidal geometry at the Ti(III) atom in **1** is caused by electronic rather than steric effects.

Spectroscopic characterization of **1** was not provided in the original report due to the extreme sensitivity of the crystals.^[4]^ We also note that **1** is difficult to handle. Non‐crystalline samples of **1** are oils at room temperature and even drying crystals of **1** under reduced pressure (*ca*. 0.01 Torr) for approximately 15 minutes did not prevent “sweating.” Nevertheless, the stability of **1** as a crystalline solid at room temperature was sufficient to allow the product to be brought into a dry box and prepared for analysis purposes. The thermal stability of crystalline **1** is limited to *ca*. 57 °C, whereas solutions of **1** show signs of rapid decomposition even at room temperature (see SI). A color change from blue to green is apparent within 15 min after the initial reaction mixture is filtered or when solid **1** is dissolved for spectroscopic analyses. For comparison, isoelectronic Ti{N(SiMe_3_)_2_}_3_ is stable at room temperature both as a solid and in hydrocarbon solutions.^[8]^

The ^1^H NMR spectrum of **1** in toluene‐*d*_8_ (see SI) shows two resonances that are assignable to the ligand methine C*H* (−0.37 ppm) and methyl Si(C*H*_3_)_3_ protons (0.06 ppm). A solution of **1** was prepared in a glovebox and frozen in liquid nitrogen but even with this precaution, decomposition was evident in the ^1^H NMR spectrum. Consequently, measuring the effective magnetic moment accurately *via* the Evans’ method proved impossible. However, the paramagnetic nature of **1** permitted analysis by EPR and hyperfine sublevel correlation (HYSCORE) spectroscopies (see SI). The continuous wave (CW) X‐band (9.4 GHz) EPR spectrum of a frozen solution (toluene) sample of **1** was collected at 30 K (Figure 2), demonstrating an axial *S*=1/2 signal consistent with the Ti(III) oxidation state assignment. Though low in abundance, the spin‐active isotopes of Ti (^47^Ti, *I*=5/2, 7.44 %; ^49^Ti, *I*=7/2, 5.41 %) contribute to resolvable hyperfine interactions (Figure 2, inset). Overall, the spectrum can be well simulated using the following parameters: *g*=[1.998, 1.857, 1.857] and |*A|* ^47/49^Ti=[101, 67, 67] MHz. These values are similar to those reported for the isoelectronic Ti{N(SiMe_3_)_2_}_3_.^[8]^

**Figure 2:**
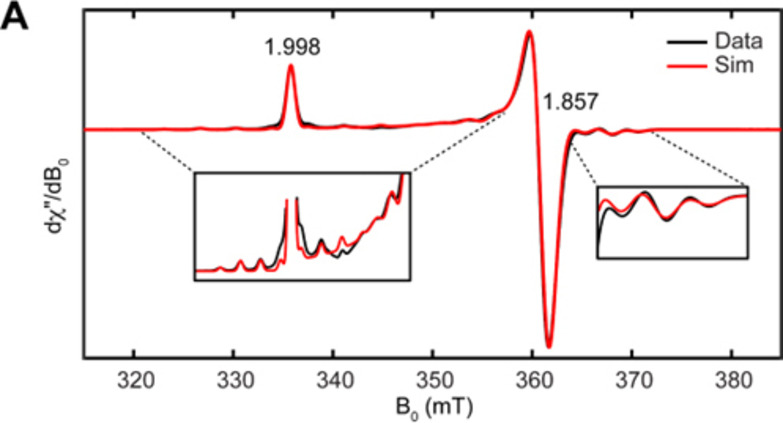
CW X‐band EPR spectrum of **1** with experimental data in black traces and simulation in red traces. Regions of the spectrum displaying Ti hyperfine interactions are shown in insets. Experimental conditions: T=30 K, microwave power=0.2 mW, modulation amplitude=0.5 mT.

Interestingly, warming the frozen solution sample of **1** from 30 K to 200 K within the cryostat resulted in the generation of a new doublet in the CW X‐band EPR spectrum (see SI), centered at about *g*=2.00241 (free electron *g* value=2.00232) with hyperfine features attributable to coupling to ^29^Si (4.68 %). The spectrum can be well simulated using a single large hyperfine coupling to a hydrogen nucleus with *a*_iso_^1^H (*I*=1/2)=48.6 MHz and smaller couplings to two equivalent silicon nuclei with *a*_iso_^29^Si (*I*=1/2)=33.7 MHz. Based on the *g*‐value and the relatively narrow linewidth of the signal, we assign it to the organic ⋅CH(SiMe_3_)_2_ radical generated during the decomposition of **1** (see below). In agreement with this assignment, the key hyperfine coupling constants calculated for ⋅CH(SiMe_3_)_2_ are *a*_iso_^1^H (*I*=1/2)=−50.9 MHz and *a*_iso_^29^Si (*I*=1/2)=38.0 MHz. Significantly smaller calculated couplings were found for the other H nuclei in **1**, which, thereby, contribute only to the spectral linewidth.

Complex **1** was also characterized *via* its electronic absorption spectrum in hexane (see SI) which features one absorption at 675 nm with a molar absorption coefficient ϵ=86 M^−1^ cm^−1^. For comparison, Ti{N(SiMe_3_)_2_}_3_ features two absorptions in the visible region at 349 and 574 nm.^[8]^ Thus, the λ_max_ of **1** is shifted bathochromically with respect to its bis(trimethylsilyl)amide congener. This may place the alkyl ligand ‐CH(SiMe_3_)_2_ lower in the spectrochemical series than ‐N(SiMe_3_)_2_, but more detailed studies are required to fully assess their properties.

Due to the instability of **1** in solution at room temperature, the reaction to form **1** was repeated on a larger scale to determine if any decomposition product could be isolated and characterized. We found that trace quantities of amber colored crystals could be grown from the amber supernatant liquid when a solution of **1** in hexane was left to stand at room temperature overnight. Analysis of the crystals by X‐ray crystallography revealed the structure of the product as the dimeric species [{(Me_3_Si)_2_HC}Ti{*μ*‐CHSiMe_2_CHSiMe_3_}]_2_ (**2**) with inversion symmetry and half a molecule per asymmetric unit (Figure 3, see SI for details). In **2**, a methyl group from one ‐CH(SiMe_3_)_2_ ligand has been activated and there is a complete loss of a ‐CH(SiMe_3_)_2_ ligand from each metal center. This activated product supports the formation of the radical species that was observed in the EPR spectroscopic study, and suggests that the principal decomposition product of **1** is the ⋅CH(SiMe_3_)_2_ radical while **2** is formed as a minor product. The activated carbon centers C14 and C14′ bridge the titanium atoms to form a central four‐membered ring and carry a single hydrogen atom each (Figure 3). Thus, the core arrangement of **2** features three non‐coplanar four‐membered rings, resulting in a ladder‐type structure with one terminal ‐CH(SiMe_3_)_2_ group at each end.

**Figure 3:**
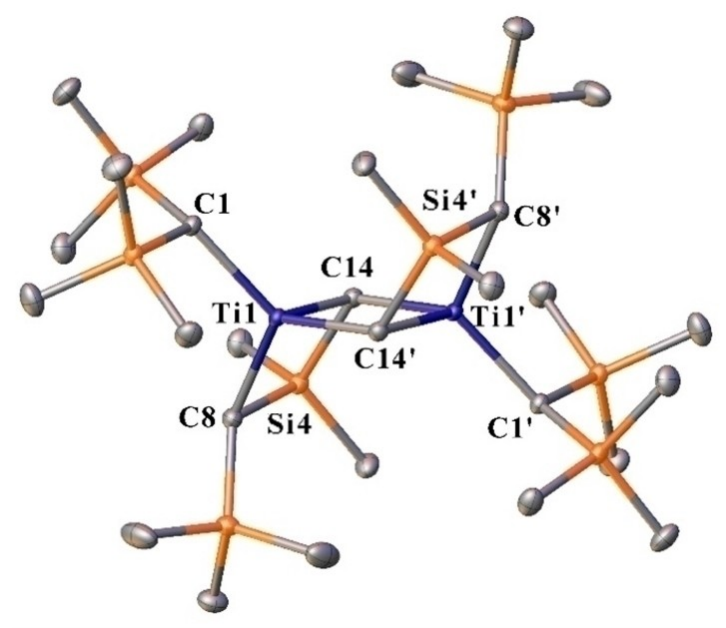
Thermal ellipsoid plot (50 %) of [{(Me_3_Si)_2_HC}Ti{*μ*‐CHSiMe_2_CHSiMe_3_}]_2_ (**2**). with hydrogen atoms not shown for clarity. Selected distances (Å) and angles (°) with calculated values in square brackets: Ti1‐C1 2.1030(8) [2.074], Ti1‐C8 2.0644(9) [2.042], Ti1‐C14 2.0035(8) [1.997], Ti1‐C14′ 2.0818(9) [2.058], Si4‐C8 1.8933(9) [1.890], Si4‐C14 1.8928(9) [1.885], Ti1⋅⋅⋅Ti1′ 2.9371(3) [2.931], C1‐Ti1‐C8 115.68(3) [114.6], C8‐Ti1‐C14 89.57(3) [90.2], C8‐Ti1‐C14′ 118.30(3) [119.4], C8‐Si4‐C14 98.39(4) [98.5], C14‐Ti1‐C14′ 88.08(3) [87.4], Ti1‐C14‐Ti1′ 91.92(3) [92.6].

The Ti1⋅⋅⋅Ti1′ distance in **2** is 2.9371(3) Å, which is significantly less than the sum of van der Waals radii for two titanium atoms (*ca*. 3.74 Å). For comparison, the covalent radius deduced for Ti from crystallographic data is 1.6 Å,^[13]^ whereas the theory‐based Pyykkö‐Atsumi single bond radius is slightly shorter, 1.36 Å.^[14]^ Thus, the distance between Ti1 and Ti1′ in **2** hints at the possibility of a covalent Ti−Ti interaction. However, calculations performed for **2** reproduce the experimental structure (Figure 3) and show that the complex has a closed shell singlet ground state with both metal atoms void of *d*‐electrons, that is, an oxidation state of +IV. In a similar vein, the results from bonding analyses do not reveal any strong electronic interaction between the metals, implying that the short Ti1⋅⋅⋅Ti1′ distance in **2** arises purely from the restrictions imposed by the ligands. Thus, compound **2** has two Ti(IV) ions, despite the vibrant color of the crystals. A computational analysis of the electronic properties of **2** showed that the first excited singlet state has a maximum at 423 nm in the gas phase, which matches well with the amber color of **2**. The transition is formally parity forbidden (g→g) but can easily become allowed if the center of symmetry is eliminated, for example, by an asymmetric vibration.

Binuclear titanium complexes with covalent Ti−Ti bonds are scarce.^[15]^ The similarity of the structure of **2** to some of the structurally characterized examples prompted further investigations on the topic. Specifically, the diamagnetic Ti(III) formamidinate complex (CyNC(H)NCy)_4_Ti_2_Cl_2_ ⋅ 2THF features a Ti⋅⋅⋅Ti distance of 2.942(2) Å, which is identical with that in **2**, and a possibility for a Ti−Ti single bond was raised and supported on the basis of results from unrestricted HF/STO‐3G calculations.^[16]^ A reanalysis of the electronic structure of the formamidinate complex using modern density functional theory methods showed that it has two Ti(III) centers in an open shell singlet diradical ground state with a calculated Ti⋅⋅⋅Ti distance of 3.027 Å (see SI for details). If the system is treated as a closed shell singlet in the calculations, the Ti⋅⋅⋅Ti distance decreases to 2.770 Å and an analysis of the associated Kohn‐Sham orbitals imply the existence of a Ti−Ti bond with two spin‐paired electrons. Incidentally, the Ti−Ti distance calculated for such hypothetical structure is in excellent agreement with the sum of Pyykkö‐Atsumi single bond radii for two Ti atoms.^[14]^ These results strongly suggest that the range of electronic states for binuclear titanium complexes is broader than heretofore realized and open shell singlet coupling of two TI(III) centers is a viable alternative to a Ti−Ti bonding interaction.

A computational analysis of the reactivity of **1** showed that an intramolecular C−H activation can liberate CH_2_(SiMe_3_)_2_, a potential source of the radical ⋅CH(SiMe_3_)_2_, with an associated barrier of 94 kJ mol^−1^ at room temperature. Although the exact details of the subsequent reaction steps are unknown, the other product {(Me_3_Si)_2_HC}Ti{CH_2_SiMe_2_CHSiMe_3_} could easily form a dimer, from which **2** can be obtained *via* hydrogen abstraction. Although the amber colored crystals of **2** were formed only in trace quantities, they proved remarkably stable. Crystals isolated for X‐ray crystallography and not used for the initial data collection retained their amber color under a layer of Paratone oil even after 24 hours at room temperature. A crystal selected from the batch and subjected to an X‐ray analysis showed no signs of decomposition, giving unit cell parameters identical to those determined during the initial data collection.

In conclusion, we have successfully isolated and characterized the elusive, synthetically relevant Ti(III) trialkyl complex Ti{CH(SiMe_3_)_2_}_3_ (**1**). We have also identified reasons for the instability of this species as it readily undergoes an intramolecular C−H activation involving a methyl group on one of the ‐CH(SiMe_3_)_2_ ligands to form the Ti(IV) dimer [{(Me_3_Si)_2_HC}Ti{*μ*‐CHSiMe_2_CHSiMe_3_}]_2_ (**2**) as one of its decomposition products.

## Supporting Information

Spectroscopic data for **1** (^1^H NMR, UV‐Vis, IR, and EPR) and crystallographic data tables for **1** and **2**.^[17]^ Full computational details and optimized coordinates and energies of all calculated structures. Images are provided for the reaction progression to afford **1**, the bulk sample of crystalline **1**, and crystals of **1** and **2** that were selected for analysis by X‐ray crystallography. The authors have cited additional references and photos of the samples within the Supporting Information.[^18^, ^19^, ^20^, ^21^, ^22^, ^23^, ^24^, ^25^, ^26^, ^27^, ^28^, ^29^, ^30^, ^31^, ^32^, ^33^, ^34^, ^35^]

## Conflict of Interests

The authors declare no conflict of interest.

## Supporting information

As a service to our authors and readers, this journal provides supporting information supplied by the authors. Such materials are peer reviewed and may be re‐organized for online delivery, but are not copy‐edited or typeset. Technical support issues arising from supporting information (other than missing files) should be addressed to the authors.

Supporting Information

## Data Availability

The data that support the findings of this study are available from the corresponding author upon reasonable request.
