# Supplementary material for: Characterization of the Titanium(III) Tris(alkyl) Ti{CH(SiMe3)2}3 and its Conversion to a Dimeric Alkyl‐Bridged Titanium(IV) Species
Source: Chemistry. 2025 Jan 28;31(13):e202404696. doi: 10.1002/chem.202404696 (PMC11874668; doi:10.1002/chem.202404696)
Supplement: Supplementary file 1 — Supporting Information [file CHEM-31-e202404696-s001.pdf]

Chemistry–A European Journal

Supporting Information

Characterization of the Titanium(III) Tris(alkyl) Ti{CH(SiMe$_3$)$_2$}$_3$ and its Conversion to a Dimeric Alkyl-Bridged Titanium(IV) Species

Connor P. McLoughlin, Anthony J. Witt, Anni Maijala, Angela A. Shiau, Guodong Rao, R. David Britt,* Heikki M. Tuononen,* and Philip P. Power*

Supporting Information for

Characterization of the Titanium(III) Tris(alkyl) Ti{CH($SiMe_3$)$_2$}$_3$ and its Conversion to a Dimeric Alkyl-bridged Titanium(IV) Species

Connor P. McLoughlin,‡a Anthony J. Witt,‡a Anni Maijala,[b] Angela A. Shiau,[a] Guodong Rao,[a] R. David Britt,*a Heikki M. Tuononen,*b and Philip P. Power*a

[a] Department of Chemistry, University of California, One Shields Avenue, Davis, CA 95616, United States.

[b] Department of Chemistry, NanoScience Centre, University of Jyväskylä, P.O. Box 35, FI-40014, Jyväskylä, Finland.

Table of Contents

**Experimental Section**	2
General Considerations	2
X-Ray Crystallography	2
EPR Spectroscopy	2
Synthesis of Ti{CH($SiMe_3$)$_2$}$_3$ (**1**)	3
Synthesis of [{(Me_3Si)$_2$HC}Ti{μ-CH$\overline{SiMe_2C}$HSiMe$_3$}]$_2$ (**2**)	3
**Photos of Ti{CH($SiMe_3$)$_2$}$_3$** (**1**)	4
**Figure S1.** Reaction mixture of **1** in hexane at ca. −78 °C.	4
**Figure S2.** Solution of **1** in hexane at ca. 25 °C before filtration.	4
**Figure S3.** Bulk sample of crystalline **1**.	4
**Figure S4.** Crystalline **1** under a microscope in Paratone oil.	5
**Spectroscopic Data for Ti{CH($SiMe_3$)$_2$}$_3$** (**1**)	6
**Figure S5**. X-band (ESE)-EPR spectrum and pseudomodulated X-Band EPR spectrum of **1**.	6
**Figure S6**. Full X-band HYSCORE spectra of **1**.	7
**Figure S7.** Field-dependent X-band HYSCORE spectra of **1**.	8
**Figure S8.** Overlaid plots of simulated data and experimental data of **1**.	8
**Figure S9.** X-band CW EPR spectra of **1** at 200 K.	9
**Figure S10.** UV-Vis spectrum of **1**.	9
**Figure S11.** ^{1}H NMR spectrum of **1**.	10
**Figure S12**. IR spectrum of **1**.	10
**Photos of [{(Me_3Si)$_2$HC}Ti{μ-CH$\overline{SiMe_2C}$HSiMe$_3$}]$_2$** (**2**)	11
**Figure S13.** Crystalline sample of **2**.	11
**Figure S14.** Crystals of **2** from the decomposed solution of **1**.	11
**Crystallographic Data Tables**	12
**Table S1.** Crystal data and structure refinement for **1** and **2**	12
**Computational Details**	13
**Optimized Coordinates**	14

Experimental Section

General Considerations

All manipulations were carried out under anaerobic and anhydrous conditions by using standard Schlenk techniques or in a Vacuum Atmospheres OMNI-Lab drybox under an atmosphere of dry argon or nitrogen. Solvents were dried by the method of Grubbs and co-workers,[18] stored over potassium or sodium, and then degassed by the freeze-pump-thaw method. All physical measurements were carried out under strictly anaerobic and anhydrous conditions. $TiCl_3(NMe_3)_2$ and $LiCH(SiMe_3)_2$ were prepared by literature procedures.[19,20] Melting points of samples in flame-sealed capillaries were determined using a Meltemp II apparatus equipped with a partial immersion thermometer and a device limit of 250 °C. IR spectra were recorded as Nujol mulls between CsI plates on a PerkinElmer 1430 spectrometer. Electronic spectra were recorded as dilute hexane solutions in 3.5 mL quartz cuvettes using an Olis 17 modernized Cary 14 UV-Vis-near-IR spectrophotometer. NMR spectra were recorded on a Bruker 300 MHz Avance Neo spectrometer, and the ^{1}H NMR spectra were referenced to the residual solvent signals in deuterated toluene.

X-ray Crystallography

Crystals of **1** and **2** suitable for X-ray crystallographic studies were obtained from saturated Et_2O solution at ca. −18 °C and hexane solution at 25 °C, respectively. Suitable crystals were selected, mounted on a nylon cryoloop, and then placed in the cold nitrogen stream of the diffractometer. Data for **1** and **2** were collected at 100(2) K with Mo $K\alpha_1$ radiation ($\lambda = 0.71073$ Å) using a Bruker D8 Venture dual source diffractometer in conjunction with a CCD detector. The collected reflections were corrected for Lorentz and polarization effects and for absorption by using Blessing's method as incorporated into the program SADABS.[21,22] The structures were solved by direct methods and refined with the SHELXTL (2012, version 6.1) or SHELXTL (2013) software packages.[23] Refinement was carried out via full-matrix least-squares procedures, with all carbon-bound hydrogen atoms included in calculated positions and treated as riding atoms. The thermal ellipsoid plots were drawn using OLEX2 software.[24]

EPR Spectroscopy

Samples were prepared as toluene solutions (ca. 2.5 mM) and rapidly cooled in liquid nitrogen to form a frozen glass. EPR spectroscopy was performed at the CalEPR center at the Department of Chemistry, University of California, Davis. X-band CW EPR spectra were acquired on a Bruker Biospin EleXsys E500 spectrometer with a super high Q resonator (ER4122SHQE) in perpendicular mode. Temperature control was achieved using liquid helium and an Oxford Instruments (Oxford, UK) ESR-900 cryogen flow cryostat and an ITC-503 temperature controller. Pulse X-band experiments were carried out on the Bruker Biospin EleXsys E580 spectrometer using a MS5 split-ring resonator. Temperature control was achieved using liquid helium and an Oxford Instruments (Oxford, UK) CF395 cryogen flow cryostat. The following pulse sequence was used for HYSCORE experiments: $\pi/2-\tau-\pi/2-t_1-\pi-t_2-\pi/2-\tau-$echo. Eight-step phase cycling was used. Time-domain spectra were baseline corrected using a third order polynomial, apodized with a hamming window, zero-filled to 8-fold points, and then fast Fourier transformed to generate

the frequency-domain spectra. Spectra were simulated using EasySpin release 6.0.2 with Matlab R2022b.[25]

Synthesis of $Ti\{CH(SiMe_3)_2\}_3$ (1)

0.104 g (0.381 mmol) of $TiCl_3(NMe_3)_2$ and 0.190 g (1.142 mmol) of $LiCH(SiMe_3)_2$ were combined in a 50 mL Schlenk flask. The flask was cooled to ca. −78 °C using a dry ice/acetone bath and ca. 40 mL of hexanes were added via cannula over ca. 10 minutes. The dry ice/acetone bath was removed, and the flask was warmed to room temperature to give a blue solution with a grey precipitate. The solution was stirred at room temperature for ca. 20 minutes and then filtered with a filter-tipped cannula. The hexanes were removed under reduced pressure to afford a blue oil which was dried under reduced pressure for ca. 20 minutes. The addition of ca. 5 mL of diethyl ether gave a blue solution that was stored at ca. −18 °C overnight to afford large blue-green needles of $Ti\{CH(SiMe_3)_2\}_3$ (**1**). Hydrocarbon and diethyl ether solutions of **1** are not stable at room temperature with a notable color change from blue to green occurring after ca. 15 min. If left to stand at ca. 25 °C, hydrocarbon solutions of **1** become an amber color overnight. If the scale of the reaction is sufficient, these decomposed solutions of **1** afford crystals of $[\{(Me_3Si)_2HC\}Ti\{\mu\text{-}CH\overline{SiMe_2C}HSiMe_3\}]_2$ (**2**). Solutions of **1** decompose after 2 weeks at ca. −18 °C, after 3 weeks at ca. −35 °C, and after 4 weeks at ca. −78 °C. Crystalline **1** was stored in the absence of solvent at temperatures below ca. −18 °C but it is sufficiently stable at room temperature for ca. 1h. However, the crystals of **1** "sweat" at room temperature. Yield: 0.06 g (32 %). Melting point: 57 °C (dec.). ^1H NMR (300 MHz, C_7D_8, δ/ppm): 0.06, -0.37. UV-vis (2.5 mM, hexane, λ/nm, ($\varepsilon/M^{-1}cm^{-1}$)): 675 (86). IR (CsI, Nujol, $\tilde{\nu}/cm^{-1}$): 2960(s), 2920(s), 2860(s), 1460(m), 1380(w), 1250(s), 1100(m), 1050(m), 1020(m), 920(w), 845(s), 800(s), 690(w), 660(w), 610(w).

Synthesis of $[\{(Me_3Si)_2HC\}Ti\{\mu\text{-}CH\overline{SiMe_2C}HSiMe_3\}]_2$ (2)

0.149 g (0.545 mmol) of $TiCl_3(NMe_3)_2$ and 0.272 g (1.635 mmol) of $LiCH(SiMe_3)_2$ were combined in a 50 mL Schlenk flask. The flask was cooled to ca. −78 °C using a dry ice/acetone bath and ca. 40 mL of hexanes were added via cannula over ca. 10 minutes. The dry ice/acetone bath was removed, and the flask was warmed to room temperature to give a blue solution with a grey precipitate. The flask was stirred at room temperature for ca. 20 minutes and then filtered with a filter-tipped cannula. The blue hexane solution was concentrated to ca. 10 mL under reduced pressure and left to stand at room temperature. Trace quantities of amber colored crystals of **2** were observed to form on the wall of the flask overnight and were removed from the Schlenk flask and immediately covered in Paratone oil for X-ray crystallographic analysis. Crystalline **2** is stable for ca. 24 h. under ambient conditions if covered in Paratone oil. Samples of **2** are X-band EPR silent.

Photos of Ti{CH(SiMe$_3$)$_2$}$_3$ (1)

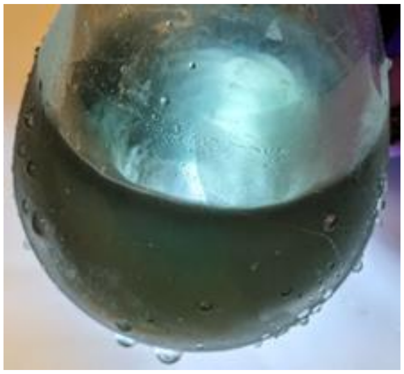

Figure S1. Reaction mixture of **1** in hexane at ca. −78 ^{o}C.

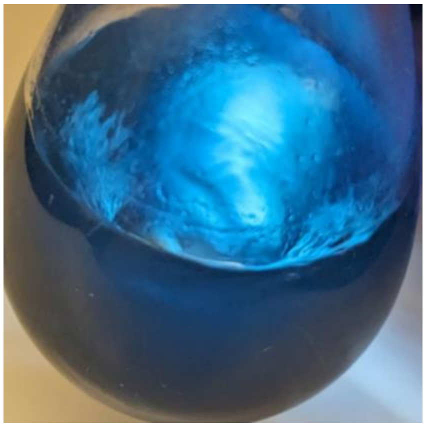

Figure S2. Solution of **1** in hexane at ca. 25 ^{o}C before filtration.

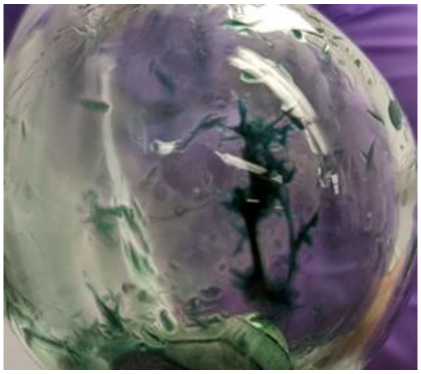

Figure S3. Bulk sample of crystalline **1**.

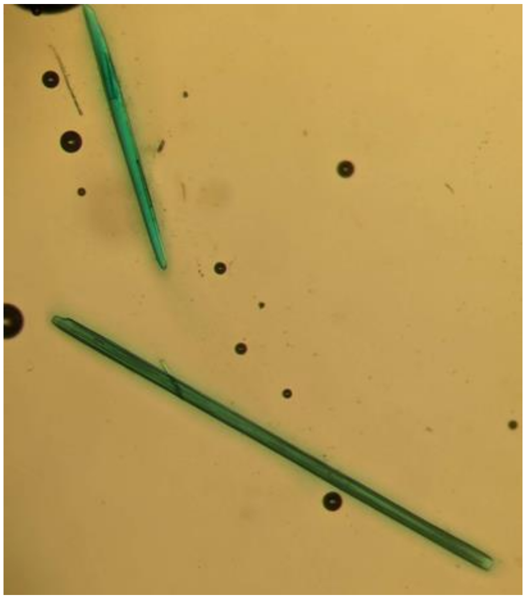

Figure S4. Crystalline **1** under a microscope in Paratone oil.

Spectroscopic Data for Ti{CH(SiMe$_3$)$_2$}$_3$ (1)

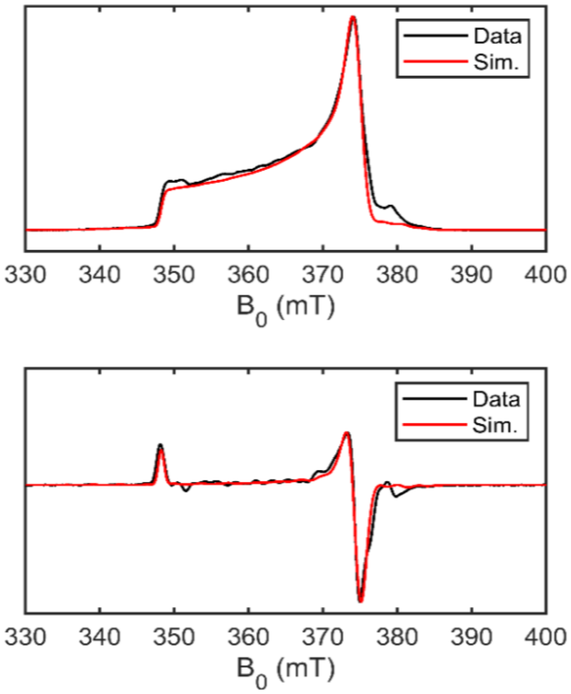

Figure S5. Top: X-band electron spin echo (ESE)-EPR spectrum of **1** (black trace) and simulation (red trace). **Bottom:** Pseudomodulated X-band ESE-EPR spectrum of **1** (black trace) and simulation (red trace) using modulation amplitude = 1 mT. Acquisition parameters: T = 30 K, frequency = 9.738 GHz, $\pi/2$ pulse = 12 ns, τ = 300 ns. Simulation parameters: g = [1.998, 1.857, 1.857], A $^{47/49}$Ti = [101, 67, 67] MHz.

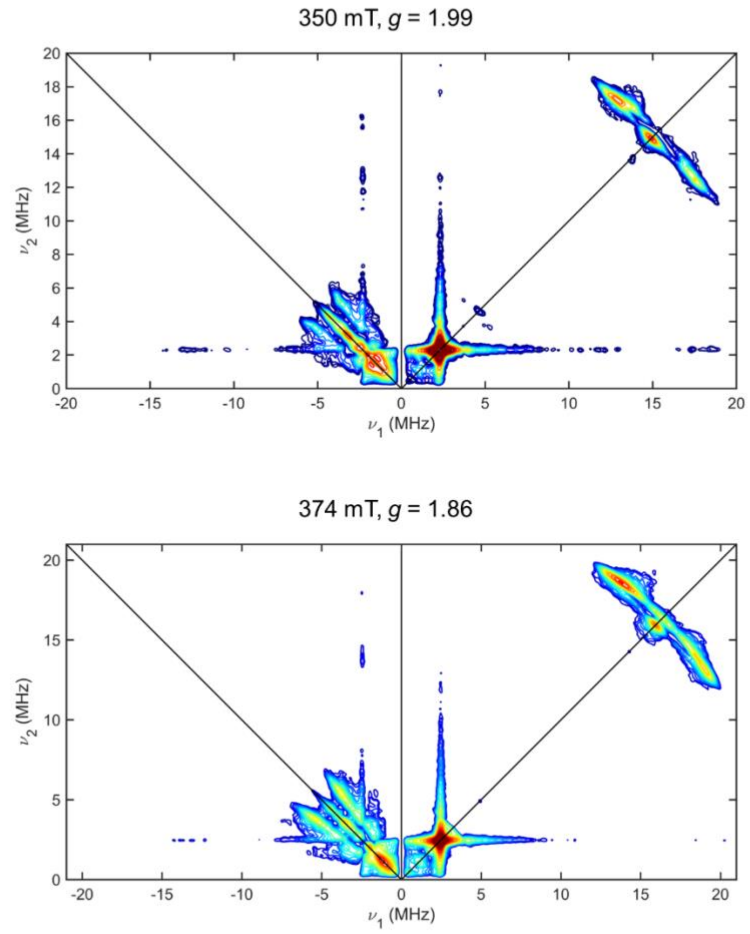

Figure S6. Full X-band HYSCORE spectra of **1** collected at 350 mT (top) and 374 mT (bottom). Sample was prepared in deuterated toluene and major features centered about ^{2}H Larmor frequency (2.29 MHz at 350 mT, 2.44 MHz at 374 mT) are due to matrix ^{2}H. Acquisition parameters: T = 30 K, frequency = 9.738 GHz, τ = 136 ns, $t_1 = t_2$ = 84 ns, $\Delta t_1 = \Delta t_2$ = 20 ns, shot repetition time (srt) = 1 ms.

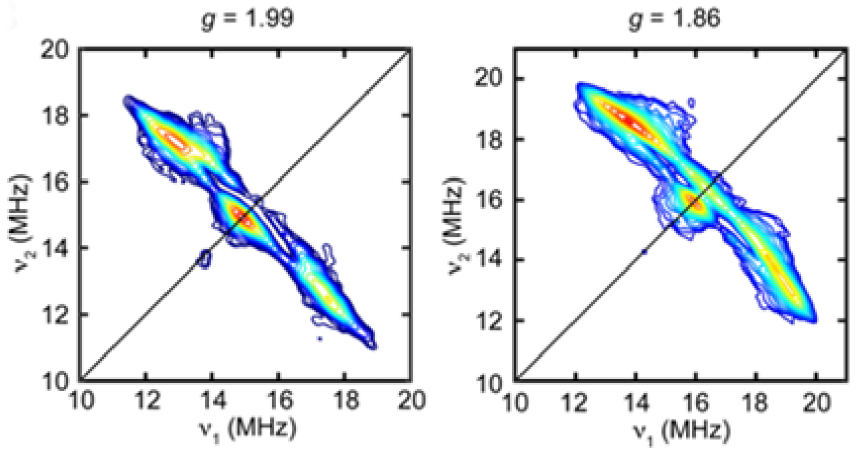

Figure S7. Field dependent X-band HYSCORE spectra of **1** collected at g = 1.99 (350 mT) and g = 1.86 (374 mT). Sharp, non-simulated signals directly overlapping with the diagonal line at the 1H Lamor frequency (14.9 MHz at 350 mT, 15.9 MHz at 374 mT) could correspond to matrix protons.

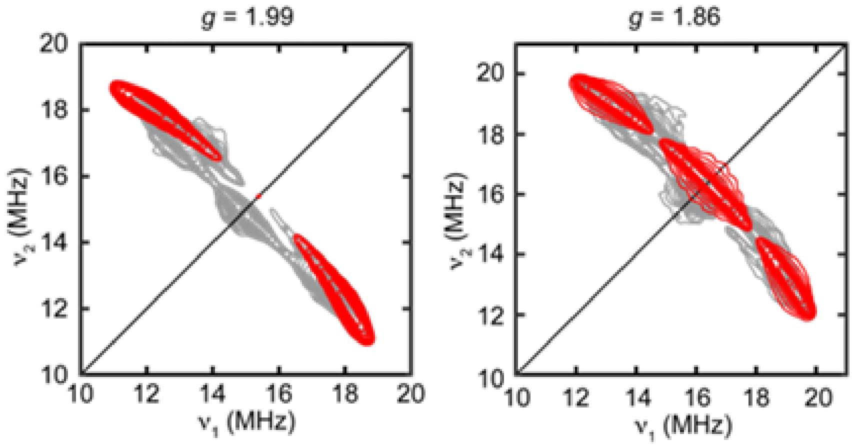

Figure S8. Overlaid plots of simulated data (red traces) and experimental data (gray traces). Experimental conditions: T = 30 K, frequency = 9.738 GHz, τ = 136 ns, t1 = t2 = 84 ns, Δ t1 = Δ t2 = 20 ns, shot repetition time (srt) = 1 ms. See text for simulation parameters and additional information.

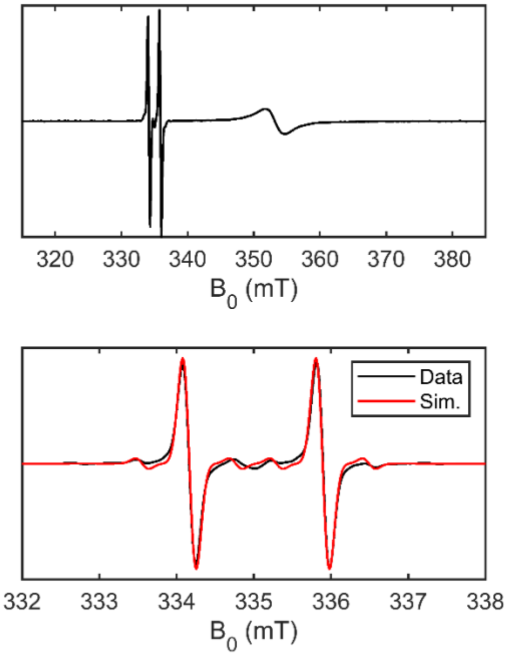

Figure S9. Top: X-band CW EPR spectrum of **1** warmed to 200 K (solution), indicating decomposition, and generation of an organic radical along with a broad, higher field feature attributable to a Ti containing species. **Bottom:** Zoomed-in spectrum of organic decomposition product (black trace) with simulation (red trace). Acquisition parameters: T = 200 K, frequency = 9.4 GHz, microwave power = 0.1 mW, modulation amplitude = 0.1 mT. Simulation parameters: g = 2.00241, A ^{1}H = 48.6 MHz, and 2 × A ^{29}Si = 33.7 MHz.

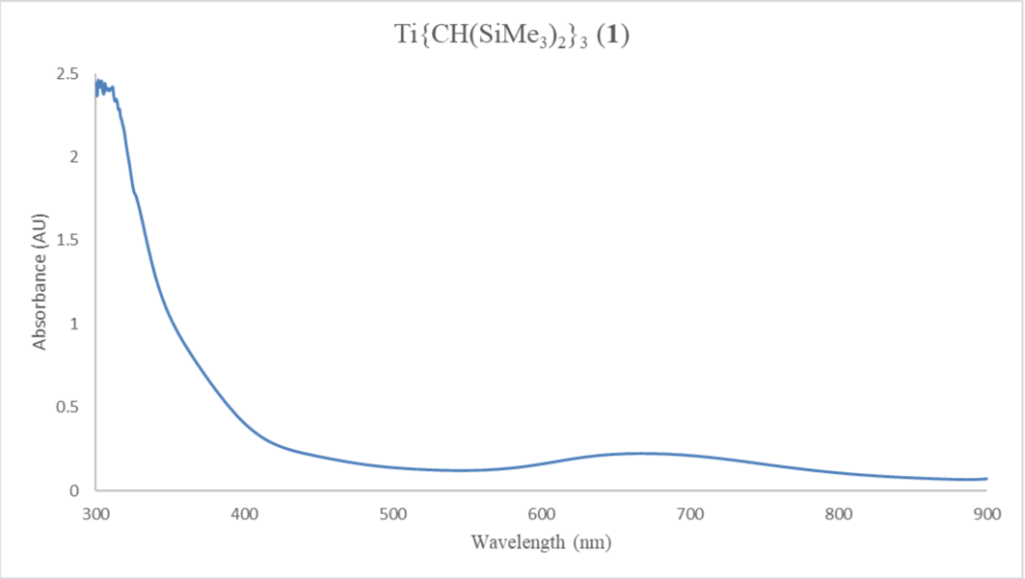

Figure S10. UV-Vis spectrum of **1** (2.5 mM in hexane) at 25 ^{o}C.

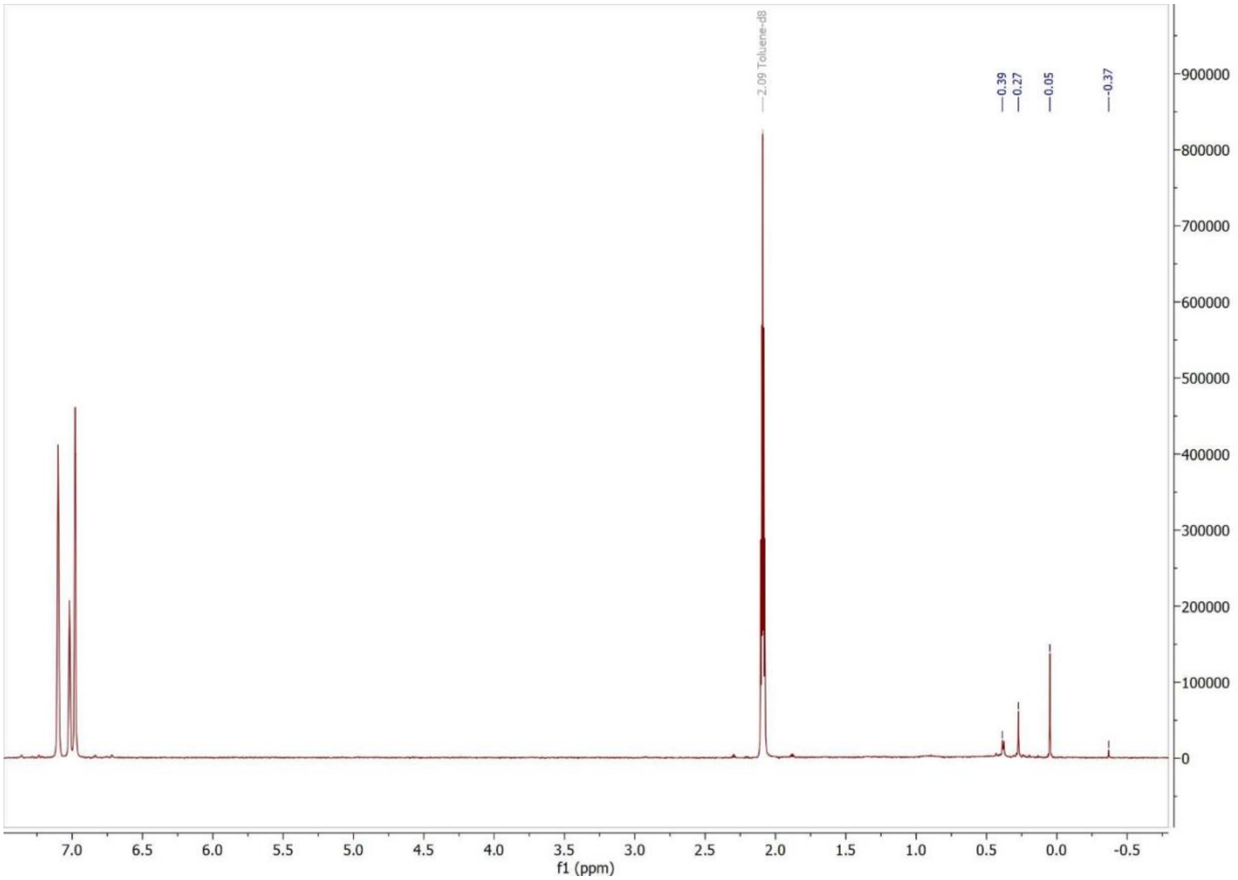

Figure S11. ^{1}H NMR spectrum of **1** (300 MHz, C_7D_8) at 25 °C. Silicone grease contaminant (0.27 ppm) and a decomposition product (0.39 ppm) are shown.

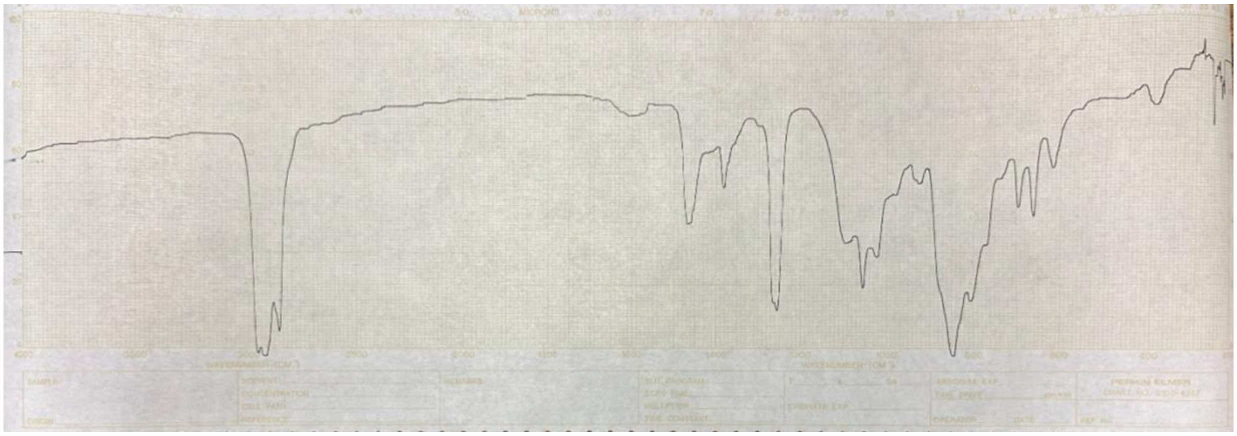

Figure S12. IR spectrum of **1** (Nujol, CsI).

Photos of [{($Me_3Si)_2$HC}Ti{μ-CH$\overline{SiMe_2C}$HSiMe$_3$}]$_2$ (2)

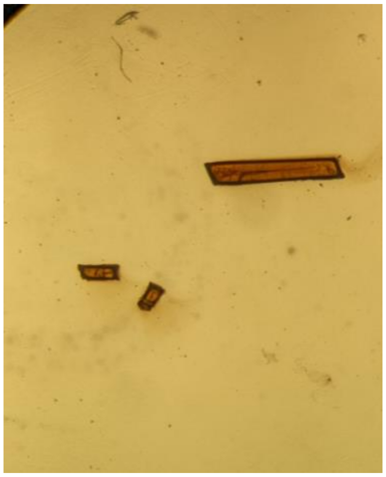

Figure S13. Crystalline sample of **2**.

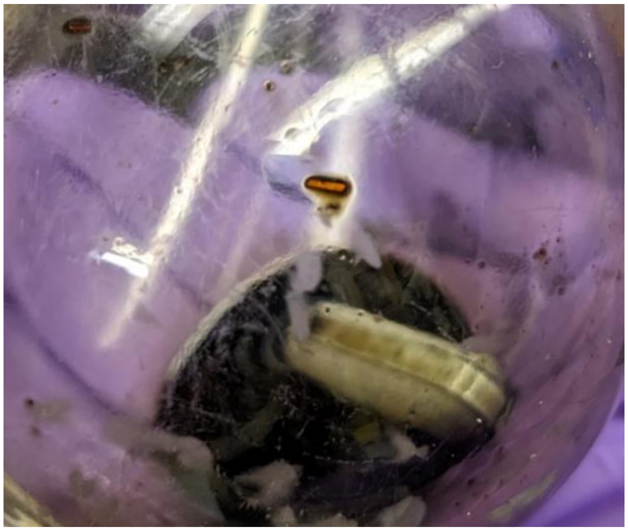

Figure S14. Crystals of **2** from the decomposed solution of **1**.

Crystallographic Data Tables

Table S1. Crystal Data and Structure Refinement for **1** and **2**

Compound	(**1**)	(**2**)
Empirical formula	C21 H57 Si6 Ti	C28 H72 Si8 Ti2
Formula weight	526.10	729.37
Temperature	100(2) K	100(2) K
Wavelength	0.7107 Å	0.7107 Å
Crystal system	Trigonal	Triclinic
Space group	*P*31c	*P*-1
Unit cell dimensions	*a* = 16.1737(5) Å	*a* = 9.1882(8) Å
	b = 16.1737(5) Å	*b* = 10.9510(9) Å
	c = 8.6470(4) Å	*c* = 11.5960(10) Å
	α = 90°	α = 91.315(2)°
	β = 90°	β = 111.932(2)°
	γ = 120°	γ = 99.925(2)°
Volume	1958.91(15) $Å^3$	1061.27(16) $Å^3$
Z	2	1
Density (calculated)	0.892 Mg m^{-3}	1.141 Mg m^{-3}
Absorption coefficient	0.408 mm^{-1}	0.619 mm^{-1}
F(000)	578	396
Crystal size	0.748 x 0.082 x 0.079 mm^3	0.260 x 0.239 x 0.150 mm^3
Crystal color and habit	blue rectangular	orange rectangular
Theta range for data collection	2.518 to 27.488°.	2.812 to 30.522°.
Index ranges	−20<=*h*<=20, −21<=*k*<=18, −9<=*l*<=11	−13<=*h*<=13, −15<=*k*<=15, −16<=*l*<=16
Reflections collected	10682	12692
Independent reflections	2876 [*R*(int) = 0.0225]	6461 [*R*(int) = 0.0119]
Observed reflections ($I > 2\sigma(I)$)	2770	6082
Completeness to theta = 25.241°	99.90 %	99.80 %
Absorption correction	None	Empirical, SADABS (multi-scan)
Max. and min. transmission	0.4598 and 0.4075	0.9011 and 0.8487
Solution method	SHELXT 2014/5 (Sheldrick, 2014)	SHELXT 2014/5 (Sheldrick, 2014)

Refinement method	SHELXL-2016/6 (Sheldrick, 2016) Full-matrix least-squares on F^2	SHELXL-2016/6 (Sheldrick, 2016) Full-matrix least-squares on F^2
Data / restraints / parameters	2876 / 1 / 92	6461 / 0 / 187
Goodness-of-fit on F^2	1.086	1.05
Final *R* indices [$I>2\sigma(I)$]	$R1$ = 0.0213, $wR2$ = 0.0545	$R1$ = 0.0205, $wR2$ = 0.0565
R indices (all data)	$R1$ = 0.0224, $wR2$ = 0.0550	$R1$ = 0.0221, $wR2$ = 0.0577
Absolute structure parameter	0.07(3)	N/A
Largest diff. peak and hole	0.212 and −0.118 e Å^{-3}	0.356 and −0.362 e Å^{-3}

Computational Details

Geometries of **1** and **2** were optimized in the gas phase with dispersion corrected density functional theory, namely the PBE1PBE functional,[26–29] def2-TZVP basis sets[30] and Grimme's D3 correction with Becke-Johnson damping,[31,32] using the Gaussian 16-C.01 program suite.[33] The structures were confirmed to be minima on the potential energy hypersurface *via* calculation of the associated vibrational frequencies (all positive). Natural Bond Orbital (NBO) analyses were performed with the NBO 7.0.5 code.[34] Vertical excitation energies and associated oscillator strengths were calculated for **2** with the time-dependent density functional theory (TD-DFT), as implemented in the Gaussian program package,[35] using the same functional-basis set combination as with prior geometry optimization.

Second-order perturbative analysis of Fock matrix in the NBO basis revealed a range of donor-acceptor interactions in **1**, the strongest of which are up to 20 kJ mol^{-1} and involve donation from C_α–Si_β, Si_β–C_γ, or C_α–H bonds to the lone vacant or Rydberg orbitals at the metal. Repeating the NBO analysis for $\mathbf{1}^-$ and $\mathbf{1}^+$ showed that the key donor-acceptor interactions weaken in the series $\mathbf{1}^+ > \mathbf{1} > \mathbf{1}^-$, which correlates with the trend in the sum of bond angles calculated for Ti (322.1, 349.1, and 357.5º, respectively). For comparison, the calculated structures of $[Ti\{N(SiMe_3)_2\}_3]^+$ and $[Ti\{O\text{-}C_6H_3\text{-}2,6\text{-}^tBu_2\}_3]^+$ (isoelectronic with $\mathbf{1}^+$) are distinctively pyramidal, but those of $Ti\{N(SiMe_3)_2\}_3$ and $Ti\{O\text{-}C_6H_3\text{-}2,6\text{-}^tBu_2\}_3$ (isoelectronic with **1**) as well as $[Ti\{N(SiMe_3)_2\}_3]^-$ and $[Ti\{O\text{-}C_6H_3\text{-}2,6\text{-}^tBu_2\}_3]^-$ (isoelectronic with $\mathbf{1}^-$) are all planar. Thus, all systems with an empty *d*-orbital at Ti, that is, oxidation state +IV. are pyramidal owing to stabilizing orbital interactions between the ligands and the metal, as discussed previously by Kaupp.[] In the case of $-NR_2$ and -OR ligands, addition of only one *d*-electron leads to repulsion between the Ti(III) center and the lone pair(s) at the ligands, such that their geometries become planar. The situation is vastly different for the $-C(H)R_2$ ligand owing to its C–H moieties, leading to a small stabilizing interaction with Ti(III) and Ti(IV), and thereby to pyramidal geometries. An estimate of the strength of these interactions could be obtained by calculating the torsional bending potential of **1**, which showed that planarization requires only 11 kJ mol^{-1}, that is, less than 4 kJ mol^{-1} (1 kcal mol^{-1}) per C_α–H···Ti interaction.

Further support for the above explanation was obtained by optimizing the structure of $Ti\{C(Me)(SiMe_3)_2\}_3$. With no possibility for C_α–H···Ti interactions, the complex has planar geometry at the metal. In contrast, the geometry of $TiMe_3$ is pyramidal at the metal and similar to that of **1**, whereas that of $Ti(SiH_3)_3$ is planar alike that of $Ti\{C(Me)(SiMe_3)_2\}_3$. Thus, the pyramidal geometry at the Ti(III) center in **1** is caused by electronic rather than steric effects.

Optimized Coordinates

85
[Ti{CH(SiMe3)2}3]+ (1+)

Ti	0.000000000311	-0.000000000277	-0.552183822805
Si	2.969140203769	0.779693095872	1.240674293798
Si	2.634579786014	-1.333982371604	-1.174701531243
C	3.293680541949	-2.940748232940	-0.494305073937
H	3.575123552823	-3.615559965165	-1.307351161031
H	2.574370592890	-3.458131009090	0.140807545913
H	4.187692355954	-2.752095562749	0.103986852192
C	1.998706625774	1.597797659734	2.623858782213
H	1.429878381197	2.474944833957	2.307431432098
H	2.705396108334	1.941189642201	3.384891390602
H	1.312912013056	0.899209606538	3.110467056889
C	4.250576725129	-0.332076189174	2.032186704821
H	3.787824822070	-1.124293763458	2.626249736251
H	4.890466183855	0.251555137924	2.699895498988
H	4.898306728996	-0.801903529274	1.288383248693
C	1.835983282713	-0.294138661961	0.180607687096
H	1.429526720456	-1.052837524433	0.881074178446
C	3.988631097698	-0.455477273376	-2.110306003733
H	4.876489490790	-0.327081143760	-1.488228623531
H	3.699303754960	0.525332957245	-2.490915346304
H	4.278128184412	-1.071279234985	-2.966853600868
C	3.844121095752	2.081617842659	0.217516887804
H	4.714906142794	1.667265073492	-0.292598491802
H	4.202031518866	2.879202874146	0.874683977197
H	3.205856306710	2.541360516087	-0.537907575341
C	1.253289898470	-1.701354528911	-2.429895711691
H	1.636888548638	-2.457648130514	-3.122253996773
H	0.991127642656	-0.848732483485	-3.065017367369
H	0.344870558826	-2.145292656507	-2.005716722733
Si	-2.159804129838	2.181504295240	1.240674293798
Si	-0.162027270770	2.948604208102	-1.174701531243
C	0.899922405113	4.322785137063	-0.494305073937
H	1.343605002554	4.903927800310	-1.307351161031
H	1.707644007269	3.958535836059	0.140807545913
H	0.289538493233	5.002695744179	0.103986852192
C	-2.383086676096	0.932031882081	2.623858782213
H	-2.858304289543	0.000838584775	2.307431432098
H	-3.033817597649	1.372346935331	3.384891390602
H	-1.435194368890	0.687410352287	3.110467056889
C	-1.837701946521	3.847145518598	2.032186704821
H	-0.920245450336	3.842499402042	2.626249736251
H	-2.663086231595	4.109490381920	2.699895498988
H	-1.754684536535	4.643009826791	1.288383248693

C	-0.663260087636	1.737077494049	0.180607687096
H	0.197020682216	1.764425216835	0.881074178446
C	-1.599860659032	3.681994492934	-2.110306003733
H	-2.154984165573	4.386704351507	-1.488228623531
H	-2.304603563672	2.941024548803	-2.490915346304
H	-1.211309059935	4.240607305155	-2.966853600868
C	-3.724794480362	2.288297602130	0.217516887804
H	-3.801346979656	3.249595958688	-0.292598491802
H	-4.594478590865	2.199464605083	0.874683977197
H	-3.803810920234	1.505672743765	-0.537907575341
C	0.846771293873	1.936058154152	-2.429895711691
H	1.309941440497	2.646411130856	-3.122253996773
H	0.239460070615	1.282707957991	-3.065017367369
H	1.685442659902	1.371312992529	-2.005716722733
Si	-0.809336072996	-2.961197391943	1.240674293798
Si	-2.472552514310	-1.614621837329	-1.174701531243
C	-4.193602946127	-1.382036904954	-0.494305073937
H	-4.918728554443	-1.288367835976	-1.307351161031
H	-4.282014599225	-0.500404827799	0.140807545913
H	-4.477230848253	-2.250600182261	0.103986852192
C	0.384380051257	-2.529829542645	2.623858782213
H	1.428425909280	-2.475783419563	2.307431432098
H	0.328421490249	-3.313536578363	3.384891390602
H	0.122282356768	-1.586619959655	3.110467056889
C	-2.412874777674	-3.515069330255	2.032186704821
H	-2.867579370799	-2.718205639414	2.626249736251
H	-2.227379951326	-4.361045520675	2.699895498988
H	-3.143622191527	-3.841106298347	1.288383248693
C	-1.172723194143	-1.442938832918	0.180607687096
H	-1.626547401738	-0.711587693233	0.881074178446
C	-2.388770437732	-3.226517220389	-2.110306003733
H	-2.721505324283	-4.059623208577	-1.488228623531
H	-1.394700190354	-3.466357506879	-2.490915346304
H	-3.066819123543	-3.169328071000	-2.966853600868
C	-0.119326614456	-4.369915445620	0.217516887804
H	-0.913559162204	-4.916861033011	-0.292598491802
H	0.392447072933	-5.078667480059	0.874683977197
H	0.597954614458	-4.047033260683	-0.537907575341
C	-2.100061191409	-0.234703626071	-2.429895711691
H	-2.946829988200	-0.188763001173	-3.122253996773
H	-1.230587712336	-0.433975475336	-3.065017367369
H	-2.030313217793	0.773979663148	-2.005716722733

85

Ti{CH(SiMe3)2}3 (1)

Ti	-0.000029163698	0.000295776969	-0.181546840994

Si	3.073148103876	0.358713909129	1.338085155771
Si	2.737233321999	-1.427261553874	-1.273861813298
C	3.625592828354	-3.015693927815	-0.807525986398
H	4.082704716478	-3.463289127437	-1.695217023059
H	2.947346863271	-3.755939421496	-0.377615105041
H	4.417981891052	-2.826368537681	-0.080573784222
C	2.230551544934	0.681075432645	2.985733142174
H	1.268701587862	1.184176822224	2.865982082092
H	2.862555066098	1.312552164299	3.616863178103
H	2.049474585761	-0.249901383020	3.529129961282
C	4.673753359518	-0.552748169144	1.723766691169
H	4.472106540682	-1.532932080753	2.163969132415
H	5.269449190467	0.019264312556	2.441369460256
H	5.287413367723	-0.705990519278	0.832804896568
C	1.949176317925	-0.620519799240	0.219381972720
H	1.584253137214	-1.468929772695	0.834741402746
C	3.991272284640	-0.331079011094	-2.139588261425
H	4.837021530904	-0.090142449622	-1.491743853927
H	3.562467572578	0.605532669396	-2.499895271407
H	4.385680386864	-0.866821517784	-3.008200135749
C	3.531422861237	2.013163447328	0.579625081203
H	4.035169995294	1.899518800955	-0.382175180906
H	4.207223442829	2.554139546060	1.248008484737
H	2.652365712432	2.640740616386	0.424007523321
C	1.416053654826	-1.887180547337	-2.538266086449
H	1.842908323356	-2.504857074308	-3.334142534226
H	0.974110557402	-1.007607975675	-3.015634510640
H	0.613215591885	-2.466916786193	-2.074835867140
Si	-1.849401731998	2.481094287786	1.338545240535
Si	-0.133271171355	3.084864252162	-1.271905925003
C	0.795649695269	4.649897727184	-0.805076983286
H	0.955470457789	5.269154615363	-1.692926109772
H	1.775448288101	4.433969557603	-0.373567573444
H	0.233883662634	5.241433104486	-0.079377379356
C	-1.707285466458	1.590621107968	2.986492517348
H	-1.658494760499	0.506186098632	2.867010215799
H	-2.571693831487	1.819630182241	3.616451451162
H	-0.812196284078	1.902179474654	3.531045940921
C	-1.863263172401	4.323063109849	1.723828062248
H	-0.914403058170	4.640293650161	2.164514568599
H	-2.657352001165	4.551947620709	2.440791187191
H	-2.037897209597	4.930476779120	0.832539259821
C	-0.437897544290	1.998266103221	0.220975552444
H	0.478839200243	2.106752028725	0.836915270296
C	-1.709325677416	3.620183538124	-2.139932469203
H	-2.341676414705	4.233653242370	-1.494320725008

H	-2.305570029283	2.779500064647	-2.498652528764
H	-1.442007845502	4.227412400472	-3.009929489480
C	-3.510094408355	2.048895263298	0.578519957003
H	-3.661960769909	2.540425210602	-0.384327065636
H	-4.317616878664	2.364696381646	1.245061436398
H	-3.613691251430	0.973550623944	0.424538487174
C	0.928271429690	2.172269396805	-2.535144559411
H	1.246933027628	2.850510477347	-3.332367735240
H	0.390289299769	1.346889916537	-3.010768770180
H	1.833279128052	1.770996905723	-2.071188862926
Si	-1.224000066252	-2.840343023150	1.339283360947
Si	-2.604166234731	-1.656762443461	-1.272626357150
C	-4.424126899626	-1.632794119785	-0.806620807491
H	-5.040043133346	-1.805019777390	-1.694473435811
H	-4.726474831779	-0.675362127101	-0.376820588599
H	-4.656384424489	-2.413717532439	-0.079733230354
C	-0.523164028152	-2.271107430430	2.986601433808
H	0.392341290105	-1.687886364203	2.866844599451
H	-0.290385758870	-3.134061002177	3.617189808252
H	-1.239837846679	-1.650445021391	3.530645235916
C	-2.812196771583	-3.772675674700	1.726848712250
H	-3.560867007362	-3.108948077277	2.167478362443
H	-2.612618717327	-4.573795866341	2.444766095452
H	-3.252040721254	-4.229008894233	0.836813725097
C	-1.511517776403	-1.377438387271	0.220776948053
H	-2.063413562869	-0.636964028023	0.836178338617
C	-2.280679063929	-3.290086360433	-2.139520759862
H	-2.493953853242	-4.143650780799	-1.492305328133
H	-1.255145550878	-3.385881260830	-2.500186278147
H	-2.941897642172	-3.363663734810	-3.008106398815
C	-0.019807238393	-4.063562571837	0.580248487195
H	-0.369254256527	-4.441863810301	-0.382297646808
H	0.110245809805	-4.920080268073	1.247761526948
H	0.963330724433	-3.615852661012	0.426067647100
C	-2.342592069628	-0.282470986166	-2.536865901741
H	-3.090860944517	-0.344125140344	-3.332767579423
H	-1.359769805671	-0.338879382982	-3.014115758205
H	-2.444009448372	0.702639738551	-2.073643190899

85

[Ti{CH(SiMe3)2}3]- (1-)

Ti	0.000000000311	-0.000000000277	0.038535922051
Si	3.150866930140	0.299219918224	1.308363781485
Si	2.754435216205	-1.516153716015	-1.250248487055
C	3.784865214567	-3.032132505212	-0.771904564772
H	4.192965195126	-3.521033357559	-1.662816950460

H	3.171471546702	-3.767507372031	-0.242991888838
H	4.617991804516	-2.763534834830	-0.118622430556
C	2.405535141703	0.562779789942	3.018650287547
H	1.425897660680	1.042005484897	2.953134899632
H	3.060908612199	1.180722889598	3.641832291489
H	2.261631364945	-0.394964373987	3.527497962168
C	4.835915496652	-0.508311704258	1.627208500364
H	4.714791214910	-1.501336079665	2.069317009888
H	5.426747157906	0.099972178776	2.320026957441
H	5.414606422737	-0.623204886018	0.706729605550
C	2.024611806990	-0.693479017753	0.236309726194
H	1.643618338487	-1.496383246503	0.902247698941
C	3.910420961392	-0.384386116466	-2.218925378317
H	4.746066126751	-0.030084723595	-1.610009570388
H	3.381592738945	0.486496716362	-2.611194996533
H	4.328032100637	-0.934239455348	-3.068452135510
C	3.546102369671	1.997045685204	0.590881415772
H	3.998094429150	1.919609917496	-0.400715139170
H	4.251298411031	2.524251980368	1.241159742540
H	2.650409134145	2.614370370951	0.499279205957
C	1.488747890343	-2.177629452048	-2.478995474794
H	1.985564281984	-2.710360647930	-3.297998602010
H	0.880989618163	-1.370838139003	-2.895177614412
H	0.811837779221	-2.878046699844	-1.984828753330
Si	-1.834565515343	2.579120845648	1.308363781485
Si	-0.064189973763	3.143487727634	-1.250248487055
C	0.733471170098	4.793855677636	-0.771904564772
H	0.952821737883	5.391731054258	-1.662816950460
H	1.677021320000	4.630328612154	-0.242991888838
H	0.084295469175	5.381065633910	-0.118622430556
C	-1.690149165450	1.801864646755	3.018650287547
H	-1.615352050917	0.713860854212	2.953134899632
H	-2.552990323094	2.060463171343	3.641832291489
H	-0.788766500783	2.156112402346	3.527497962168
C	-1.977746899170	4.442181522100	1.627208500364
H	-1.057200422619	4.833797004800	2.069317009888
H	-2.799952025218	4.649714808589	2.320026957441
H	-2.167591948087	5.000789155908	0.706729605550
C	-0.411735456902	2.100104765847	0.236309726194
H	0.474096736253	2.171606857822	0.902247698941
C	-1.622322338747	3.578716949605	-2.218925378317
H	-2.346978928249	4.125256194920	-1.610009570388
H	-2.112114884473	2.685296858313	-2.611194996533
H	-1.354940948542	4.215305474535	-3.068452135510
C	-3.502543480513	2.072491893268	0.590881415772
H	-3.661478168256	2.502646382940	-0.400715139170

H	-4.311715545840	2.419606432152	1.241159742540
H	-3.589315722990	0.988136454432	0.499279205957
C	1.141508480558	2.378108218207	-2.478995474794
H	1.354459033760	3.074729432325	-3.297998602010
H	0.746685843999	1.448378458617	-2.895177614412
H	2.086542665960	2.142095489794	-1.984828753330
Si	-1.316301413863	-2.878340764703	1.308363781485
Si	-2.690245241507	-1.627334012449	-1.250248487055
C	-4.518336383731	-1.761723173254	-0.771904564772
H	-5.145786932075	-1.870697697529	-1.662816950460
H	-4.848492865768	-0.862821240954	-0.242991888838
H	-4.702287272757	-2.617530799910	-0.118622430556
C	-0.715385975319	-2.364644437527	3.018650287547
H	0.189454391171	-1.755866339940	2.953134899632
H	-0.507918288171	-3.241186061772	3.641832291489
H	-1.472864863228	-1.761148029190	3.527497962168
C	-2.858168596548	-3.933869818672	1.627208500364
H	-3.657590791357	-3.332460925965	2.069317009888
H	-2.626795131754	-4.749686988196	2.320026957441
H	-3.247014473715	-4.377584270721	0.706729605550
C	-1.612876349154	-1.406625748924	0.236309726194
H	-2.117715073806	-0.675223612149	0.902247698941
C	-2.288098621711	-3.194330833969	-2.218925378317
H	-2.399087197568	-4.095171472156	-1.610009570388
H	-1.269477853538	-3.171793575506	-2.611194996533
H	-2.973091151160	-3.281066020018	-3.068452135510
C	-0.043558888223	-4.069537579303	0.590881415772
H	-0.336616259960	-4.422256301266	-0.400715139170
H	0.060417135743	-4.943858413351	1.241159742540
H	0.938906589779	-3.602506826213	0.499279205957
C	-2.630256369967	-0.200478766989	-2.478995474794
H	-3.340023314810	-0.364368785226	-3.297998602010
H	-1.627675461228	-0.077540320444	-2.895177614412
H	-2.898380444247	0.735951209219	-1.984828753330

82
[Ti{N(SiMe3)2}3]+

Ti	0.000236889994	-0.000124048356	0.576799926330
Si	2.575537297916	1.269144192809	1.093825339727
N	1.194742559824	1.308936931405	-0.005147773445
C	2.096555540299	0.011538626512	2.430174569345
H	1.913041889883	-1.012550394459	2.095441136637
H	1.293734189910	0.346510800573	3.094361029482
H	2.979172494782	-0.056778698792	3.074795540265
C	4.175773124233	0.776303564585	0.278808104947
H	4.657416324542	1.650600560731	-0.162256471788

H	4.047357230717	0.033797437420	-0.506876278655
H	4.864087247806	0.365468680201	1.022728708746
C	2.822040635601	2.891818774969	1.978914630096
H	3.184533456066	3.667945553078	1.302663821546
H	3.585204158756	2.753889268127	2.750953792138
H	1.918387202174	3.262457574255	2.466130874176
Si	1.067785452268	2.645501793549	-1.171587068608
C	-0.167516468854	2.221270944580	-2.497223541612
H	-1.151917150812	1.995183169808	-2.090799204267
H	0.169263820557	1.365199975057	-3.084373256709
H	-0.265674655149	3.071271432265	-3.178075111891
C	0.586947678667	4.220317743650	-0.277605232836
H	1.463924411185	4.710098102892	0.148686186274
H	-0.128613656802	4.061051105379	0.528932762140
H	0.135643429057	4.922681994836	-0.984101519085
C	2.725763556746	2.942715507040	-1.981148044551
H	3.478522596388	3.306602356507	-1.278962789973
H	2.593806593243	3.721799904389	-2.738414737572
H	3.123340242081	2.060487433978	-2.485848175860
N	0.536632973806	-1.689390035278	-0.004781979000
Si	-0.187818605671	-2.865947655203	1.093698498219
C	-1.040167204329	-1.823853046451	2.429480228627
H	-1.837468029530	-1.155716527737	2.094321965550
H	-0.350065507130	-1.293194905317	3.092860962561
H	-1.537813123689	-2.554838959856	3.075385247958
C	-1.412652399257	-4.007143553248	0.277733564220
H	-0.895750267552	-4.866042227686	-0.153573152673
H	-1.984221534513	-3.528440298180	-0.515546240666
H	-2.119241550485	-4.390705521120	1.019077810662
C	1.095005112004	-3.888998433445	1.979727144070
H	1.588462337300	-4.589408153841	1.303672819708
H	0.593681525561	-4.482689385740	2.750281328937
H	1.865722228831	-3.290749805102	2.469096147621
Si	1.756347514844	-2.247231478795	-1.172678825431
C	2.005714105434	-0.965260112257	-2.498443745643
H	2.300540513672	0.000873732179	-2.092060181893
H	1.096375908855	-0.830195156470	-3.086597689444
H	2.792006575353	-1.304426393813	-3.178460896376
C	3.361771176586	-2.618559198002	-0.281014590527
H	3.346886629174	-3.622064786499	0.147348633701
H	3.585119468092	-1.917926507663	0.523404563900
H	4.193940326542	-2.581812075413	-0.989758848326
C	1.183958614941	-3.831811793275	-1.981413626678
H	1.122755479766	-4.665161456151	-1.278645660492
H	1.923958859235	-4.107802407920	-2.739075858011
H	0.220853443022	-3.734934453127	-2.485553908590

N	-1.730901234081	0.379635707640	-0.004637375340
Si	-2.386640242742	1.596340158287	1.093227298952
C	-1.058564034316	1.812421925262	2.429840218063
H	-0.079865757513	2.165801063827	2.095568571610
H	-0.947422644890	0.950449477203	3.094984491770
H	-1.441491751938	2.611436830140	3.073414025456
C	-2.760427953767	3.227729256949	0.276929080286
H	-3.756771102714	3.205579432474	-0.167744748764
H	-2.050903157492	3.489361098615	-0.506113618738
H	-2.753243006908	4.029138300725	1.021042916399
C	-3.915095282130	0.998863471281	1.979076792954
H	-4.768815461649	0.924280201846	1.303215056317
H	-4.177070877976	1.729180188314	2.750766801610
H	-3.784055817488	0.031284310235	2.466950950623
Si	-2.824418101402	-0.398006377001	-1.171921405921
C	-1.839554721953	-1.253360660714	-2.499285951197
H	-1.149054764978	-1.991069962056	-2.093884536601
H	-1.269391066031	-0.532642502104	-3.087922573234
H	-2.526590997285	-1.765439563994	-3.178590662062
C	-3.946104095206	-1.604334537791	-0.279764107508
H	-4.802775276050	-1.090071448091	0.158779234684
H	-3.446398356283	-2.155230963289	0.516867637862
H	-4.337920765602	-2.337302882079	-0.990725500801
C	-3.912117646321	0.889644973629	-1.978938265548
H	-4.604075021126	1.357342265311	-1.275743893391
H	-4.520218731923	0.387010780198	-2.737487532611
H	-3.347396792177	1.676632763604	-2.481780831853

82
Ti{N(SiMe3)2}3

Ti	0.000000000001	-0.000252264072	-0.000000000001
Si	1.011011976171	2.794942399365	1.134998339327
N	0.000000000001	1.942076305221	-0.000000000001
C	1.640681331889	1.606864498578	2.444002254923
H	2.218952278014	0.784294877331	2.019341416810
H	0.818193635013	1.184847069480	3.027393428429
H	2.289699496748	2.149940474488	3.137731782618
C	2.486263593793	3.600894969241	0.301164397925
H	2.188531718618	4.484372364866	-0.266454303701
H	2.992935870099	2.921898886546	-0.386257172662
H	3.214152376407	3.920084160435	1.053020199328
C	0.067373750697	4.147623202262	2.029062199965
H	-0.219432001463	4.960496237836	1.358080709731
H	0.703919493712	4.576315402481	2.809002248716
H	-0.838102914697	3.766790225537	2.507062055270
Si	-1.011011976169	2.794942399365	-1.134998339329

C	-1.640681331887	1.606864498578	-2.444002254925
H	-2.218952278012	0.784294877331	-2.019341416812
H	-0.818193635012	1.184847069480	-3.027393428431
H	-2.289699496747	2.149940474488	-3.137731782619
C	-2.486263593792	3.600894969241	-0.301164397927
H	-2.188531718616	4.484372364866	0.266454303699
H	-2.992935870097	2.921898886546	0.386257172660
H	-3.214152376406	3.920084160435	-1.053020199330
C	-0.067373750696	4.147623202262	-2.029062199967
H	0.219432001464	4.960496237836	-1.358080709733
H	-0.703919493710	4.576315402481	-2.809002248718
H	0.838102914699	3.766790225537	-2.507062055272
N	1.682182792613	-0.971218586604	-0.000369660647
Si	1.915273871450	-2.275358021098	1.132046729281
C	0.572338445986	-2.228909217081	2.442132441570
H	-0.429546243788	-2.317801955602	2.018244847882
H	0.618417514467	-1.306558093763	3.027021406690
H	0.718840673061	-3.063604862593	3.134419684181
C	1.875117673453	-3.954315864817	0.294857722697
H	2.788576331730	-4.137066236955	-0.274100198348
H	1.033113112511	-4.052404471117	-0.391940862505
H	1.788392490471	-4.745731596189	1.045280403624
C	3.559034213403	-2.136679704093	2.025383474405
H	4.405844475608	-2.293842062446	1.353510785294
H	3.612217726610	-2.903786998627	2.803833737068
H	3.682607347563	-1.163039327722	2.505104306782
Si	2.926812403084	-0.519632342005	-1.133706265082
C	2.212839153227	0.620229779304	-2.442295657379
H	1.788455298726	1.531505914638	-2.017064930856
H	1.437005462046	0.118778097925	-3.026722456924
H	3.007925903339	0.912143028645	-3.135161601163
C	4.360666657822	0.355832489889	-0.297831034111
H	4.976942273635	-0.343275305679	0.270423041186
H	4.024525590405	1.133736812198	0.389289370703
H	5.001474659340	0.827306515346	-1.048878150712
C	3.629134328292	-2.011536378522	-2.028314312265
H	4.189930653597	-2.666015535033	-1.357195684720
H	4.318925684543	-1.673086270331	-2.807357005712
H	2.847803273404	-2.605946556513	-2.507466552243
N	-1.682182792611	-0.971218586604	0.000369660646
Si	-2.926812403082	-0.519632342005	1.133706265081
C	-2.212839153225	0.620229779304	2.442295657377
H	-1.788455298724	1.531505914638	2.017064930854
H	-1.437005462044	0.118778097925	3.026722456922
H	-3.007925903338	0.912143028645	3.135161601162
C	-4.360666657821	0.355832489889	0.297831034109

H	-4.976942273633	-0.343275305679	-0.270423041187
H	-4.024525590404	1.133736812198	-0.389289370705
H	-5.001474659338	0.827306515346	1.048878150710
C	-3.629134328290	-2.011536378522	2.028314312264
H	-4.189930653595	-2.666015535033	1.357195684719
H	-4.318925684541	-1.673086270331	2.807357005710
H	-2.847803273403	-2.605946556513	2.507466552241
Si	-1.915273871449	-2.275358021098	-1.132046729283
C	-0.572338445984	-2.228909217081	-2.442132441571
H	0.429546243790	-2.317801955602	-2.018244847884
H	-0.618417514466	-1.306558093763	-3.027021406691
H	-0.718840673059	-3.063604862593	-3.134419684183
C	-1.875117673451	-3.954315864817	-0.294857722699
H	-2.788576331729	-4.137066236955	0.274100198346
H	-1.033113112509	-4.052404471117	0.391940862503
H	-1.788392490469	-4.745731596189	-1.045280403625
C	-3.559034213401	-2.136679704093	-2.025383474407
H	-4.405844475606	-2.293842062446	-1.353510785296
H	-3.612217726608	-2.903786998627	-2.803833737069
H	-3.682607347562	-1.163039327722	-2.505104306784

82
[Ti{N(SiMe3)2}3]-

Ti	0.000305596576	-0.000019117827	-0.001076872809
Si	2.660908795220	1.363330138469	1.143954942691
N	1.381192280288	1.452818200065	0.000055811172
C	2.406661662452	0.043579790605	2.457274024088
H	2.243487170725	-0.939894547832	2.012581811907
H	1.537737986531	0.270658483017	3.078353502598
H	3.294996114491	-0.005599820929	3.099684535087
C	4.317801204701	0.971559589948	0.325696185956
H	4.663741293343	1.794197390097	-0.303735350023
H	4.246238066836	0.082774369773	-0.305183002802
H	5.083370624625	0.783453701898	1.085434600416
C	2.923357630259	2.981886596183	2.080121277483
H	3.280751570031	3.780018656749	1.424268883384
H	3.672128154215	2.836967320036	2.865606900295
H	1.998130237673	3.321701126398	2.552548886106
Si	1.228887770644	2.726700471367	-1.143656703499
C	-0.076702195163	2.408516815716	-2.457217862147
H	-1.050909922113	2.196190251942	-2.012878572254
H	0.193278559346	1.552673108438	-3.079245344864
H	-0.169882235363	3.294064598336	-3.098615775846
C	0.756326624751	4.362281624569	-0.325171861578
H	1.561925912495	4.749331693755	0.302344257299
H	-0.126402868589	4.246566660612	0.307610814292

H	0.528555951182	5.117110124925	-1.084798803136
C	2.832745455541	3.068822371254	-2.079433415741
H	3.612100454551	3.464591028904	-1.423025443090
H	2.651435311169	3.810026622361	-2.864541964727
H	3.217754659855	2.161670901092	-2.552247312355
N	0.567134112768	-1.922632246859	-0.001191246936
Si	-0.150490931483	-2.986225360740	1.142431687146
C	-1.170131958789	-2.106710020206	2.453134276367
H	-1.940240254033	-1.474854337578	2.006811754588
H	-0.540810447469	-1.466503907843	3.074757279220
H	-1.656943109002	-2.851661874068	3.095223091585
C	-1.314198051213	-4.228232997326	0.322770223279
H	-0.771624016230	-4.941298532356	-0.301543917091
H	-2.045067332591	-3.724204776090	-0.313538215619
H	-1.863556946698	-4.794651652021	1.081746411904
C	1.119614273177	-4.020082507575	2.082139892965
H	1.634318172730	-4.728518956213	1.427833809797
H	0.619010329194	-4.596153109649	2.867113195706
H	1.874788772901	-3.387318944379	2.555460962326
Si	1.747657724681	-2.426952982859	-1.144005486398
C	2.127213867405	-1.135870514095	-2.455610919291
H	2.431128841115	-0.187028677196	-2.009636455717
H	1.251613881772	-0.939851158560	-3.077908342259
H	2.940745391480	-1.497766740246	-3.097024055680
C	3.399195943275	-2.837224612550	-0.323933141717
H	3.330133012047	-3.728302572967	0.303579301566
H	3.740571041125	-2.015211158953	0.309195538252
H	4.167366108503	-3.018166244833	-1.082785087703
C	1.242205254329	-3.985524556740	-2.082276645025
H	1.194598699880	-4.859253128804	-1.427102803028
H	1.975183378717	-4.198218897244	-2.867226820874
H	0.264397048547	-3.864440684222	-2.555491952472
N	-1.948416852485	0.469614241449	-0.000002158064
Si	-2.510538280141	1.623430629357	1.143083966940
C	-1.239789060989	2.064975193530	2.455100898090
H	-0.307201739000	2.415953250936	2.009340732038
H	-1.000524114537	1.199513423439	3.076395730971
H	-1.641473043168	2.858910163619	3.097385457387
C	-3.001300735122	3.253070912684	0.323469606522
H	-3.887989669649	3.140310342625	-0.303931977871
H	-2.197247358230	3.634556864299	-0.309778728909
H	-3.219661825314	4.011276154256	1.082496459166
C	-4.042658616615	1.041733005894	2.080835449218
H	-4.912552784794	0.949593667508	1.425303733688
H	-4.292373900562	1.764097422881	2.864718548743
H	-3.872961011670	0.071829865765	2.555412102987

Si	-2.976301207453	-0.300632916013	-1.142146421637
C	-2.048176532813	-1.270925067247	-2.456700732678
H	-1.376957610227	-2.008337653353	-2.012555116266
H	-1.442109114673	-0.608532512589	-3.078187395075
H	-2.768193841014	-1.794308676856	-3.098454476095
C	-4.153896139782	-1.529333479696	-0.322487843541
H	-4.888466049226	-1.026774723243	0.310282353877
H	-3.610163147702	-2.238926133250	0.305124121383
H	-4.697743099978	-2.100716056759	-1.081732332986
C	-4.077112063032	0.916236954120	-2.076246604039
H	-4.808672245036	1.392824429890	-1.418504388309
H	-4.629423179561	0.388127433395	-2.860271279434
H	-3.485485449636	1.703699265609	-2.550177188903

109
[Ti{O-C6H3-2,6-tBu2}3]+

Ti	-0.000163070424	0.000321817003	-0.506518430519
O	0.749219716414	1.516437402386	-0.047011730533
O	0.937920684990	-1.407313393742	-0.048363829212
O	-1.688072353119	-0.108703053288	-0.047773730923
C	1.265700089561	2.776900493788	0.001741116845
C	2.085621465387	3.138629250955	1.086582981749
C	2.405174743511	2.202891193539	2.253725784368
C	3.146112940567	0.955355299793	1.762135868971
H	2.536375505711	0.327824943165	1.117297842781
H	4.048024539134	1.234991434837	1.213223642212
H	3.448322147390	0.346741190833	2.617190865711
C	1.115049886411	1.819538785959	2.982106773763
H	0.402179361921	1.314917997942	2.334229796964
H	1.340648317231	1.150833538534	3.816033907335
H	0.624859943219	2.709227768651	3.383561481692
C	3.313253219171	2.874714739906	3.283590024240
H	3.499712451421	2.170840337235	4.096898477455
H	4.281610518979	3.151444828969	2.861100095084
H	2.854506571353	3.764327896929	3.719964764341
C	2.599563927653	4.430760658651	1.065338976544
H	3.232904216999	4.762276265378	1.874746486720
C	2.327438972430	5.309505899056	0.035710541613
H	2.747547496990	6.307788578653	0.051147167619
C	1.520705226425	4.917342278592	-1.014245158249
H	1.324557065518	5.621460599487	-1.809289048616
C	0.962165640015	3.646259406462	-1.064580110774
C	0.063765020765	3.248054698536	-2.239219130289
C	-0.024629389280	4.357358443696	-3.290251413714
H	0.953211251717	4.612376737288	-3.702884630898
H	-0.655737052653	4.018360473168	-4.114408938096

H	-0.478591785172	5.262202882487	-2.883391872238
C	-1.367328246750	2.992613285562	-1.758898687316
H	-1.768365992347	3.888132306863	-1.281367259524
H	-2.012397141719	2.743213613538	-2.604611145902
H	-1.450927070074	2.190277128529	-1.025819990355
C	0.626234908107	2.035448814661	-2.981543155174
H	0.786093462350	1.147243086870	-2.352037474711
H	-0.034942114745	1.736032299576	-3.796961595332
H	1.614034554199	2.252990661922	-3.392835355807
C	1.772015531229	-2.484284018284	-0.000489469304
C	2.676302431459	-2.654982118226	-1.067304850930
C	2.779362200680	-1.677511781292	-2.241783585027
C	1.447566137612	-1.559045802040	-2.983552738024
H	1.518169975995	-0.836461770240	-3.798796857164
H	1.142893275714	-2.523387249622	-3.395194428814
H	0.598381624869	-1.254277931365	-2.353756652879
C	3.273348025252	-0.310300612870	-1.761508928832
H	2.621210520264	0.162571913450	-1.027152894887
H	4.250206356459	-0.410306364549	-1.285526794560
H	3.378193467346	0.373451929509	-2.607089400097
C	3.784107625062	-2.154804933173	-3.293379174953
H	3.805311831100	-1.438538524290	-4.117375658099
H	4.794968164988	-2.213706333260	-2.887088241931
H	3.516360536947	-3.129164110622	-3.706192450798
C	3.498673791037	-3.773633064031	-1.017821788184
H	4.206267155469	-3.954971019216	-1.813276418301
C	3.436066020299	-4.668886185800	0.031734727770
H	4.091211210543	-5.531362351808	0.046505922351
C	2.539245892673	-4.466359684530	1.061812974797
H	2.510580030740	-5.181053457088	1.870863252925
C	1.676420958260	-3.375829578260	1.083985953315
C	0.706849753933	-3.185883487518	2.251784966477
C	0.836067445829	-4.308614789360	3.281029923048
H	1.836183853519	-4.355611189095	3.716729796689
H	0.133641179155	-4.119037704612	4.094872750363
H	0.591958790831	-5.285565046445	2.858211660733
C	-0.744312773237	-3.204353411685	1.761182788926
H	-0.952839899595	-4.124845751877	1.211504890642
H	-1.421928970859	-3.163077651094	2.616745718392
H	-0.983977390259	-2.362078360107	1.117363451838
C	1.019756393972	-1.877129576929	2.980667953909
H	0.937627634172	-1.007038796671	2.333535032566
H	0.328783082589	-1.739248593386	3.815551140489
H	2.035867250335	-1.896863731374	3.380826388072
C	-3.037739901690	-0.292634637604	0.001862363515
C	-3.760704970743	0.235649271204	1.087402962500

C	-3.110019810430	0.980159165273	2.254537425207
C	-2.132123410351	0.054701712721	2.981911317121
H	-1.665890203462	0.584081728767	3.816095205671
H	-2.656734685797	-0.815382860838	3.382824877150
H	-1.338651505725	-0.309271977169	2.333465925676
C	-2.401111351116	2.246235135410	1.763199992003
H	-3.095001668315	2.887495115509	1.215247361348
H	-2.024676154078	2.811894801552	2.618294613263
H	-1.553263602734	2.032737321001	1.117482766578
C	-4.145652482374	1.429387042413	3.285182648682
H	-4.870296188023	2.129229101291	2.863397829437
H	-4.685757538496	0.586651961902	3.721487720417
H	-3.629278850241	1.942874541904	4.098439434315
C	-5.136531556895	0.033532600708	1.067112577791
H	-5.740074443906	0.415472751410	1.877079456985
C	-5.761705290857	-0.641693649613	0.037757430882
H	-6.836165242108	-0.777961660500	0.053985218988
C	-5.019070930317	-1.143223434468	-1.012925495465
H	-5.531004918136	-1.665173864600	-1.807798137331
C	-3.639183419756	-0.990136137258	-1.064317572125
C	-2.845781918015	-1.567820810715	-2.239990741034
C	-2.077973162259	-0.473586613463	-2.982265622325
H	-1.387592391956	0.108054233017	-2.353221150334
H	-1.489318253825	-0.895748708777	-3.798966288598
H	-2.760830881096	0.273497623897	-3.391922627525
C	-1.908074701560	-2.679228643209	-1.761014388124
H	-2.482373768449	-3.475396492820	-1.284441971336
H	-1.369257301295	-3.111687850532	-2.607291533348
H	-1.171720285439	-2.350624846443	-1.027558216741
C	-3.762826557332	-2.198901763914	-3.290636232704
H	-4.473549201584	-1.479776354823	-3.701988220082
H	-3.154242990105	-2.574816039155	-4.115725115894
H	-4.318483205175	-3.045236216686	-2.884033682907

109
Ti{O-C6H3-2,6-tBu2}3

Ti	0.000135799666	0.000325518226	-0.052135755589
O	0.059730628981	-1.568301567511	-0.949646421271
O	0.001819878313	0.008557488921	1.755994273851
O	-0.062361698335	1.561277940554	-0.962758687555
C	0.105012319311	-2.735063806879	-1.616868158683
C	1.220996436136	-3.581361477315	-1.439413782525
C	2.388325853089	-3.231150302221	-0.511115826323
C	1.907321301974	-3.108498981233	0.938490906664
H	1.181877941018	-2.311695442747	1.081979963057
H	1.440254345291	-4.041039136691	1.263403835634

H	2.755359995586	-2.905526241231	1.597568295184
C	3.079441975095	-1.942947259213	-0.969426442904
H	2.407457536586	-1.087423553445	-0.989901655496
H	3.907776313231	-1.702065815972	-0.297730376581
H	3.482024633519	-2.065683494805	-1.977797745525
C	3.463459501537	-4.319087513406	-0.515836235122
H	4.271533130505	-4.016439838931	0.153989638874
H	3.079463742313	-5.276728452976	-0.157123491959
H	3.894995298231	-4.466480724125	-1.508466656568
C	1.231472333500	-4.778391297929	-2.147004786766
H	2.067522154073	-5.454448006433	-2.039867489321
C	0.197073630797	-5.136339808952	-2.988731568499
H	0.233045835282	-6.076859999346	-3.525856751196
C	-0.883319270977	-4.290425232912	-3.143025651721
H	-1.682168208504	-4.588859285926	-3.806543231155
C	-0.965184459697	-3.074939647858	-2.472493988809
C	-2.180725246659	-2.166214970155	-2.681223770191
C	-3.190561652173	-2.782702339843	-3.650532837094
H	-3.580332986456	-3.736094850307	-3.286661558085
H	-4.036058910017	-2.099390265105	-3.757720305804
H	-2.763779016050	-2.937120312984	-4.644110494874
C	-1.755735499019	-0.828732746327	-3.296376222798
H	-1.246721026566	-0.993839675795	-4.248763783980
H	-2.635488616512	-0.207960083612	-3.484543782927
H	-1.082551339959	-0.260175604779	-2.659660610457
C	-2.929673377130	-1.948870013836	-1.362496756429
H	-2.307875672542	-1.495051278437	-0.593493838134
H	-3.792055573225	-1.296470804396	-1.523792277729
H	-3.291277383521	-2.901947043091	-0.969074393952
C	0.002792597444	0.012816255165	3.100769830115
C	-1.054696454433	-0.622538102234	3.787006373959
C	-2.208578846660	-1.325139533652	3.064685594223
C	-2.988682617173	-0.334074392346	2.194990561370
H	-3.806309288318	-0.847547282885	1.681996634125
H	-3.417466168790	0.458422046651	2.813062227577
H	-2.368690097660	0.143444380010	1.438989221710
C	-1.687053472918	-2.496917741452	2.226362630185
H	-1.004771330898	-2.188062055578	1.438402668779
H	-1.155773921037	-3.210283786096	2.860599193192
H	-2.522751778408	-3.019272567515	1.753632533663
C	-3.217801299483	-1.917310443890	4.049738255597
H	-4.018800624919	-2.398824864413	3.484225244909
H	-2.766407473661	-2.676112408709	4.692900891629
H	-3.673623852438	-1.151473646101	4.681388730514
C	-1.019303527096	-0.595546453985	5.176797619962
H	-1.810273572164	-1.069357509050	5.740135227431

C	0.004776563083	0.021330406516	5.867742346550
H	0.005544523290	0.024641509080	6.951422276058
C	1.027792967244	0.634215413885	5.171644171597
H	1.819445082470	1.111450997444	5.731162606508
C	1.061276382098	0.652472208960	3.781546944891
C	2.213557689080	1.350963876901	3.052897150272
C	3.223437487731	1.950562936846	4.032680462901
H	3.680495032923	1.189369646218	4.669039370911
H	4.023499835851	2.428830500288	3.463098172562
H	2.772204958020	2.713354994054	4.671201986419
C	1.690147172464	2.516469151855	2.206915754357
H	1.159146110550	3.234023988411	2.836621471643
H	2.525086440956	3.035993548487	1.729710812753
H	1.007308691971	2.201746156534	1.421728413884
C	2.993275965711	0.354558442097	2.189033194430
H	2.372757735930	-0.128263179014	1.436855034555
H	3.809909607618	0.865176253137	1.671632232527
H	3.423357984493	-0.433514193580	2.811849760104
C	-0.108324252145	2.721888846434	-1.640521858526
C	0.960925730361	3.054194861855	-2.500401525350
C	2.177453633593	2.144930744636	-2.700830880436
C	2.927572890761	1.941366719591	-1.380559214035
H	3.791061726444	1.289075812033	-1.536284874663
H	3.287655227962	2.898724267847	-0.996236931347
H	2.307184248097	1.493611094272	-0.606887966384
C	1.753723418550	0.800983750118	-3.302464868599
H	1.244655266068	0.955963640525	-4.256531043224
H	2.633979733930	0.178988229386	-3.484080500038
H	1.080940558461	0.238386431954	-2.660035769785
C	3.185879704639	2.753495701095	-3.676628018740
H	2.759011741177	2.895895100324	-4.671958310519
H	3.572899746750	3.711868546527	-3.323077071348
H	4.033316871897	2.071442638499	-3.776118279242
C	0.877776216094	4.263183684243	-3.182354408656
H	1.675856352639	4.555708888791	-3.849410707981
C	-0.202744444771	5.110125815475	-3.034829265534
H	-0.239629457776	6.045503668308	-3.580798000689
C	-1.235902203866	4.760024012589	-2.188234960366
H	-2.071949472068	5.436919597776	-2.086456827351
C	-1.224154119521	3.569686111957	-1.469396818956
C	-2.390029106557	3.227543722708	-0.536364425814
C	-3.080783413578	1.934790637128	-0.982032694280
H	-2.408255094592	1.079538762752	-0.995278590439
H	-3.908205352542	1.699428806605	-0.307276504027
H	-3.484539665295	2.047860233036	-1.991060737883
C	-1.906978830261	3.118156638439	0.913712652207

H	-1.441143683035	4.054267466746	1.230012145070
H	-2.753962521609	2.919549728160	1.575481392212
H	-1.180135597336	2.323693378952	1.063289727793
C	-3.465591031498	4.314852091024	-0.550002948059
H	-3.898337540234	4.452542109885	-1.543493357680
H	-4.272780047133	4.018330451282	0.123618947533
H	-3.081506383170	5.276039186033	-0.201003810147

109
[Ti{O-C6H3-2,6-tBu2}3]-

Ti	-0.001250693876	0.000812147122	-0.000231785146
O	0.744357795563	1.712988980000	0.001158205441
O	1.109020541006	-1.500817475849	-0.002136875162
O	-1.856944101619	-0.209629514778	0.000498648525
C	1.274389209191	2.924665664683	0.001374126279
C	2.105986341055	3.330234186991	1.078816183167
C	2.420126514016	2.407498529219	2.261359897872
C	3.152211042373	1.147873938199	1.786409261427
H	2.549711128345	0.548325240032	1.109711588832
H	4.077398618687	1.416689627459	1.269467657693
H	3.408825644144	0.518982299452	2.643904771457
C	1.137740876579	2.029639965332	3.010224109752
H	0.457429195155	1.453410632284	2.382224848987
H	1.383417935391	1.417164398947	3.883653105535
H	0.619223945366	2.928443275229	3.355967815544
C	3.339661465333	3.082884741449	3.280571055723
H	3.530283149364	2.381035354317	4.096412511225
H	4.305070594772	3.356998909669	2.846465790047
H	2.885973510342	3.978603513640	3.713076392066
C	2.644838720430	4.611265534789	1.042837678780
H	3.281567782996	4.944967047504	1.850677576838
C	2.394220725740	5.483731402385	0.001240176961
H	2.829102688410	6.477231319883	0.001130009561
C	1.583162328333	5.075757123457	-1.040242125240
H	1.396251178381	5.769806242995	-1.848168314276
C	1.007855076169	3.810655341647	-1.076036654607
C	0.116946802533	3.414686386567	-2.258379497701
C	-0.012358918905	4.548379327614	-3.277439837426
H	0.952936923900	4.823341185918	-3.710798237623
H	-0.657812840571	4.211865477197	-4.092723071483
H	-0.466089415465	5.443308034898	-2.842898297815
C	-1.304523116650	3.096181867058	-1.782823686427
H	-1.735631108345	3.957874874095	-1.266008308703
H	-1.940667033224	2.856881000519	-2.639977672077
H	-1.334743531950	2.247054880786	-1.105727565724
C	0.710577821784	2.216947532330	-3.007478492321

H	0.750180042456	1.326154179421	-2.379545603850
H	0.094006156605	1.981162838083	-3.880761530819
H	1.722311149883	2.447024234709	-3.353453487672
C	1.896015319907	-2.563733251798	-0.002031993936
C	2.796174311800	-2.774554234179	-1.080098900426
C	2.895403085981	-1.806268301483	-2.263774114693
C	1.560350391476	-1.725033901981	-3.011487328705
H	1.662124206020	-1.073594223223	-3.885343292750
H	1.255610367791	-2.717205597576	-3.356423926466
H	0.768970712582	-1.315246745250	-2.382989586934
C	3.328515085940	-0.414514181411	-1.790946697511
H	2.608254007266	0.036286454958	-1.113925884135
H	4.290967346493	-0.469548575512	-1.275077731268
H	3.437378521986	0.254792321143	-2.649349239138
C	3.941405441584	-2.260677880323	-3.283549255105
H	3.970120050815	-1.534662950081	-4.100015652784
H	4.944020989388	-2.312497806186	-2.850325435187
H	3.698221909598	-3.235305279114	-3.715017702407
C	3.607218207082	-3.903102295696	-1.043831626343
H	4.301565690852	-4.087187506752	-1.852148133025
C	3.558478776214	-4.808532737214	-0.001374537054
H	4.203976842445	-5.680023471162	-0.001074984833
C	2.677773275548	-4.590814634690	1.040665867351
H	2.651137287758	-5.308355024056	1.849240342929
C	1.834719039713	-3.485957131983	1.076227836837
C	0.879046795517	-3.298124806706	2.259430959127
C	1.007653444177	-4.431120689235	3.279379213826
H	2.010432129684	-4.482831606773	3.711766040252
H	0.304113440317	-4.246695921111	4.095189348259
H	0.765094019512	-5.405264656290	2.845999749469
C	-0.578256712331	-3.306073549056	1.785735841051
H	-0.806392914620	-4.242808530205	1.270031117042
H	-1.250696531093	-3.214417117170	2.643695959637
H	-0.798549852303	-2.485765786807	1.108302884162
C	1.190672905443	-1.997434847963	3.007200470652
H	1.029474110215	-1.120814036918	2.378818429378
H	0.537884076211	-1.904861812210	3.881074986134
H	2.228580926818	-1.995312998419	3.352133790099
C	-3.170879496496	-0.360490473059	0.000868498262
C	-3.938916856515	0.152112570848	1.079889861446
C	-3.298307822714	0.884901353421	2.263548413004
C	-2.326897415105	-0.035613897927	3.009909170217
H	-1.919899922768	0.482775639245	3.883978852573
H	-2.843401488877	-0.936068263246	3.354363189509
H	-1.487492922345	-0.333425004257	2.380647089698
C	-2.577338547274	2.151640144216	1.790548111497

H	-3.275151185348	2.817845883439	1.276060340533
H	-2.161216676995	2.687470866754	2.648682499835
H	-1.757305758638	1.933163846979	1.112149145909
C	-4.343622745477	1.338434372077	3.284393608595
H	-5.066301583487	2.035920866524	2.852133504921
H	-4.889427433318	0.495101424240	3.715769364173
H	-3.832075435613	1.854560850010	4.100775747770
C	-5.317211166119	-0.026156802784	1.044525953068
H	-5.925267366920	0.354516057271	1.853687740435
C	-5.946059488501	-0.679271215541	0.001979930848
H	-7.023477191672	-0.803097839753	0.002433704540
C	-5.186308271832	-1.172749895430	-1.041172502592
H	-5.692840513657	-1.681465057703	-1.849889018890
C	-3.803523198311	-1.033904463821	-1.077658202751
C	-3.014644717700	-1.602230267151	-2.262199592398
C	-2.277279832988	-0.485458435476	-3.008642388766
H	-1.526510693860	-0.005755970983	-2.379771994081
H	-1.764230509184	-0.897969171605	-3.883275241480
H	-2.984418080173	0.274891600267	-3.352252331901
C	-2.025397557679	-2.673361981422	-1.790775580108
H	-2.553911672796	-3.480393190654	-1.276146846931
H	-1.499781270091	-3.100716115636	-2.649707096517
H	-1.275207167937	-2.275504606888	-1.113063847452
C	-3.931109659771	-2.280022554990	-3.282588146729
H	-4.654156848455	-1.581981688634	-3.712876467013
H	-3.316778735716	-2.666385171755	-4.099807427835
H	-4.476635615151	-3.123349339399	-2.850372222536

94

Ti{C(Me)(SiMe3)2}3

Ti	-0.007392195731	-0.007948334797	-0.387041928929
Si	0.496019865049	-3.527411651207	-0.975894085188
Si	2.149240930868	-2.086682066127	1.229455927329
C	3.934240375534	-2.479988905258	0.759572874430
H	4.525949179625	-2.572366933690	1.675254092634
H	4.408990999519	-1.709371225678	0.149190505985
H	4.011167957836	-3.423166371106	0.216319785518
C	-0.168401267773	-3.412806807717	-2.745903575947
H	-1.239977720770	-3.214707940470	-2.788809673263
H	0.000906471768	-4.377912110304	-3.231836822008
H	0.335244671743	-2.658373029196	-3.355621906771
C	1.949920395666	-4.720432448884	-1.135293804460
H	2.747927961010	-4.302447235253	-1.755208315048
H	1.606520483830	-5.639870553006	-1.618843375827
H	2.384966277180	-4.995964188452	-0.173443686349
C	1.083554917794	-1.865822820507	-0.310772503517

C	1.613770778675	-3.480814565700	2.372308429042
H	1.672761279334	-4.466520004579	1.908517709553
H	0.607944940464	-3.345607958915	2.771119596905
H	2.304482874738	-3.478990377828	3.221577694905
C	-0.806932735162	-4.412619340336	0.045953563761
H	-0.445699686408	-4.679492493105	1.039509971071
H	-1.070547087905	-5.340698617730	-0.470610355215
H	-1.723836081515	-3.837151798201	0.173708973119
C	2.212485049288	-0.544026878342	2.294970957104
H	2.998335414857	-0.641774667322	3.050372930104
H	1.267319240022	-0.376859262085	2.814644734749
H	2.428208885592	0.340778749145	1.697522683459
Si	-3.303340171590	1.323324468174	-0.991659179219
Si	-2.879807263549	-0.788259222244	1.251450592541
C	-4.116657737761	-2.145435071722	0.815008889143
H	-4.512070292032	-2.568524707508	1.743400180638
H	-3.682776321982	-2.970974511711	0.247682539657
H	-4.959359426084	-1.757367181758	0.240554629163
C	-2.871057449562	1.800454837517	-2.772161096369
H	-2.120056303976	2.588226751796	-2.839819215221
H	-3.779500525524	2.180059931855	-3.248622784461
H	-2.526065652810	0.956299285500	-3.374707090519
C	-5.067418767867	0.667303033617	-1.134752008404
H	-5.111558914303	-0.245300411784	-1.735558113577
H	-5.689788966472	1.417146639348	-1.632152574605
H	-5.521901505573	0.450796162655	-0.166646381720
C	-2.161900902636	-0.005953580984	-0.305727529128
C	-3.812765188551	0.385303062217	2.386026075872
H	-4.696935078548	0.820825570529	1.917758420781
H	-3.192977784477	1.193841147825	2.773977159367
H	-4.155437882551	-0.204111187953	3.242482793504
C	-3.409081605796	2.914446322642	-0.000510156931
H	-3.808877139539	2.753218882228	1.001191516165
H	-4.088977770028	3.594998416146	-0.522271362670
H	-2.452395791801	3.425898022656	0.106604624117
C	-1.560048670830	-1.604137997589	2.308278572573
H	-2.024418866419	-2.240608626639	3.067771712919
H	-0.943837632240	-0.864956895837	2.823211594309
H	-0.901799051705	-2.230800933551	1.706476596072
Si	2.812442944183	2.184528544480	-0.972545728575
Si	0.743174131028	2.903352777451	1.228793126217
C	0.189030881360	4.644432808471	0.754416301321
H	-0.045785413975	5.200758324121	1.666822264481
H	-0.705708797192	4.664531097632	0.129075152599
H	0.972258300653	5.188802206924	0.224515330244
C	3.039687795988	1.559399966592	-2.747037337547

H	3.388682169512	0.527585574117	-2.800081415190
H	3.801741625859	2.181447515273	-3.225195864370
H	2.137576923554	1.638748762293	-3.359037975072
C	3.117145788339	4.040771232271	-1.131295076844
H	2.350394192184	4.524579344838	-1.742678776011
H	4.080430053643	4.204110534218	-1.623796906231
H	3.147823281203	4.553312181174	-0.168622814835
C	1.083597999024	1.860661932669	-0.301272395915
C	2.214308433696	3.138737894390	2.376355275619
H	3.041993862058	3.676959530455	1.912010547458
H	2.595318512779	2.200301725815	2.780409738274
H	1.867750824682	3.741689171141	3.221715428388
C	4.239266932626	1.498347841232	0.036241853071
H	4.282262141042	1.922961909036	1.039748972354
H	5.171631044915	1.759495693645	-0.473798383283
H	4.214961815651	0.413404534455	0.139283084586
C	-0.629763887705	2.189099251192	2.289001626897
H	-0.950563970314	2.921380932737	3.036275545295
H	-0.300987968789	1.292662279215	2.817772101314
H	-1.497545644525	1.924833943295	1.685399125274
C	0.098500901062	2.224749259187	-1.427127712689
H	-0.361366271583	1.340651924425	-1.950324924017
H	-0.760615889631	2.810953194709	-1.103732461379
H	0.550017991431	2.753186776036	-2.272555114657
C	1.898987672827	-1.195371495777	-1.430250355069
H	1.379901476703	-0.336332897547	-1.936289602367
H	2.846593398948	-0.769083662585	-1.104218120505
H	2.112227749389	-1.845065147881	-2.284863177200
C	-2.019853535262	-1.081300900283	-1.404781706430
H	-1.038164588425	-1.065498744355	-1.942439272356
H	-2.111389101489	-2.105458947877	-1.045769808028
H	-2.725969286372	-0.968988235957	-2.233618345968

13

TiMe3

Ti	0.000000001224	-0.000000000408	-0.346470990974
C	1.382336091813	1.514601250222	0.022952372387
H	0.936593344568	2.346258731465	0.579251510288
H	2.251774614031	1.147915823465	0.579130147709
H	1.738694037779	1.905901863454	-0.941521306345
C	-2.002851203719	0.439837545295	0.022952372387
H	-2.519906448277	0.552802272726	-0.941521306345
H	-2.500216336100	-0.362015737993	0.579251510288
H	-2.120011570060	1.376136105943	0.579130147709
C	0.620515115579	-1.954438796741	0.022952372387
H	-0.131763040298	-2.524051930633	0.579130147709

H	0.781212414172	-2.458704137404	-0.941521306345
H	1.563622995205	-1.984242994696	0.579251510288

13
Ti(SiH3)3

Ti	-0.021646017466	0.011370462065	0.031663498978
Si	1.768546710687	1.938419476463	0.224134633916
H	1.136996242129	3.288528782365	0.341228052225
H	2.651488310060	1.762953740325	1.419325604811
H	2.671433533167	1.979734565089	-0.968392186639
Si	-2.596717782578	0.517021706642	0.027919099922
H	-3.291054712572	0.080400331287	-1.223540865733
H	-3.334914449056	-0.123341209941	1.161017864578
H	-2.820249459466	1.992552140864	0.149911286099
Si	0.793602099619	-2.467418720436	-0.166406848836
H	0.359171687539	-3.359252780137	0.953176016192
H	0.390771551110	-3.157558560274	-1.430933002467
H	2.292572296828	-2.463409954313	-0.146135753046

28
CH(SiMe3)2

Si	-2.873109404782	-2.411739439281	-1.051100257245
Si	-0.634537446047	-3.066538932477	1.324175601366
C	0.095733132991	-4.706666919978	0.769317290925
H	0.483035341121	-5.264723370126	1.626743544241
H	0.918204999261	-4.553306144706	0.066392218551
H	-0.657289658133	-5.325061089968	0.275238920549
C	-2.774754307567	-1.661365462097	-2.766992855772
H	-2.577174857989	-0.587372634178	-2.713984004203
H	-3.711958034192	-1.802440760115	-3.312414101067
H	-1.973953265085	-2.120927031039	-3.352085841082
C	-3.234564392093	-4.248638751489	-1.200406166429
H	-2.447975738201	-4.760056645131	-1.760847751200
H	-4.180264685415	-4.410519095836	-1.725270694045
H	-3.313339787111	-4.726470099174	-0.220736217754
C	-1.273059253688	-2.144795092810	-0.152721143966
C	-2.032313447120	-3.403739005089	2.532373402177
H	-2.825427904099	-4.001616624716	2.076520680737
H	-2.477651680947	-2.471726806396	2.889253137473
H	-1.661186385943	-3.953206897936	3.402091349044
C	-4.269885898987	-1.594584162176	-0.095866088558
H	-4.360811318991	-2.018110964767	0.907081721716
H	-5.223598955238	-1.738173865586	-0.612064773070
H	-4.099929540817	-0.520092357446	0.007377037767
C	0.692433671554	-2.056835273573	2.182451204661
H	1.088140062080	-2.586829840972	3.053247516421

H	0.297067531498	-1.097169268593	2.525252211743
H	1.527697859456	-1.853263554700	1.507022928503
H	-0.631469675517	-1.350049909646	-0.538262751486

110
[{(Me3Si)2HC}Ti{mu-CH(SiMe2C)HSiMe3}]2 (2)

Ti	-0.223408259220	9.202161033842	4.708252619972
Si	0.293723141293	7.648242429414	1.700058303524
Si	-2.562137766350	8.705291543450	2.630271210748
Si	-1.413611837531	7.099337212199	7.270814458772
Si	1.583968014255	7.900859835043	6.153226158988
C	-0.700453832735	8.853630335364	2.719853144880
H	-0.455735676293	9.856550276844	2.328354006346
C	0.266672487469	5.905676815091	2.402944416474
H	0.713602890697	5.848097013806	3.396948564303
H	0.842822786526	5.245219506276	1.748065753949
H	-0.746135921181	5.504688242425	2.467665159187
C	-0.354679302274	7.545588670398	-0.064720850575
H	-1.367352788332	7.137353400276	-0.108057175272
H	0.289112178546	6.899584749100	-0.668870625753
H	-0.372320481448	8.533059804266	-0.533700062637
C	2.083123076728	8.203050278730	1.583094111551
H	2.164790585362	9.188738728023	1.118346532208
H	2.657735038647	7.500219353106	0.972874201135
H	2.554868343694	8.262041472684	2.565871276508
C	-3.248154660140	9.557307585927	1.104889100967
H	-2.886965944201	9.077990517490	0.192544574402
H	-2.950885121197	10.608127498474	1.066626873758
H	-4.341292447800	9.516998681516	1.099160804353
C	-3.135442651008	6.919206472331	2.632594420128
H	-4.228117898193	6.886567447482	2.668906727993
H	-2.759986708742	6.375684506281	3.502174883749
H	-2.818260796855	6.384594415482	1.734777136512
C	-3.377222334629	9.525250973996	4.126759795652
H	-4.457484745044	9.354999756410	4.078610571789
H	-3.207693379848	10.603374790865	4.152968274466
H	-3.042131458859	9.115345169147	5.085751870015
C	-0.252877729217	7.539554911815	5.893455785788
H	-0.359185061630	6.795145327901	5.085526730205
C	-2.979241720181	6.359720659834	6.543042962960
H	-3.494749151020	7.049643516402	5.872134476162
H	-3.676355108018	6.086036899500	7.340175547018
H	-2.752281583938	5.451286672917	5.977759951518
C	-1.863356387759	8.622870016192	8.271853939888
H	-0.973885872253	9.170457338624	8.591652180832
H	-2.426566604286	8.348229885301	9.167938498112

H	-2.479871600839	9.314572413190	7.691896037584
C	-0.706142201094	5.808571616611	8.440780125624
H	-0.337897022407	4.941095498679	7.886284777932
H	-1.479492747798	5.459732294990	9.131571831402
H	0.118807303611	6.201578117046	9.037434781318
C	2.030233551833	8.041285506006	7.970540919166
H	2.985089246767	8.557894456409	8.086961477906
H	2.139698717383	7.039364353174	8.392638263639
H	1.281695761277	8.571713537154	8.560575185468
C	2.725446505109	6.617500231953	5.403884440637
H	2.598239522334	6.537930875014	4.323538487908
H	2.547623191934	5.633607960766	5.845824292477
H	3.766747899020	6.890603915805	5.597952516656
C	1.696347146805	9.503118994988	5.167358167238
H	2.549608814111	9.598154227291	4.487195112771
Ti	1.305415834978	11.344038966158	5.999283743664
Si	0.788284434464	12.897957570586	9.007478060112
Si	3.644145342108	11.840908456550	8.077265152888
Si	2.495619413288	13.446862787801	3.436721904864
Si	-0.501960438498	12.645340164957	4.554310204649
C	1.782461408493	11.692569664636	7.987683218756
H	1.537743252051	10.689649723156	8.379182357291
C	0.815335088288	14.640523184909	8.304591947162
H	0.368404685061	14.698102986194	7.310587799333
H	0.239184789231	15.300980493724	8.959470609688
H	1.828143496939	15.041511757575	8.239871204449
C	1.436686878031	13.000611329602	10.772257214211
H	2.449360364090	13.408846599724	10.815593538909
H	0.792895397212	13.646615250900	11.376406989389
H	1.454328057206	12.013140195734	11.241236426273
C	-1.001115500970	12.343149721270	9.124442252085
H	-1.082783009604	11.357461271977	9.589189831428
H	-1.575727462889	13.045980646894	9.734662162502
H	-1.472860767937	12.284158527316	8.141665087129
C	4.330162235898	10.988892414073	9.602647262669
H	3.968973519959	11.468209482510	10.514991789234
H	4.032892696955	9.938072501526	9.640909489879
H	5.423300023558	11.029201318484	9.608375559283
C	4.217450226765	13.626993527669	8.074941943508
H	5.310125473951	13.659632552518	8.038629635643
H	3.841994284500	14.170515493719	7.205361479888
H	3.900268372613	14.161605584518	8.972759227124
C	4.459229910387	11.020949026004	6.580776567984
H	5.539492320802	11.191200243590	6.628925791847
H	4.289700955605	9.942825209135	6.554568089170
H	4.124139034616	11.430854830853	5.621784493621

C	1.334885304975	13.006645088185	4.814080577848
H	1.441192637388	13.751054672099	5.622009633431
C	4.061249295938	14.186479340166	4.164493400676
H	4.576756726777	13.496556483598	4.835401887474
H	4.758362683775	14.460163100500	3.367360816618
H	3.834289159695	15.094913327083	4.729776412119
C	2.945363963517	11.923329983808	2.435682423748
H	2.055893448011	11.375742661376	2.115884182804
H	3.508574180043	12.197970114699	1.539597865525
H	3.561879176597	11.231627586810	3.015640326052
C	1.788149776852	14.737628383389	2.266756238013
H	1.419904598165	15.605104501321	2.821251585705
H	2.561500323556	15.086467705010	1.575964532234
H	0.963200272147	14.344621882954	1.670101582319
C	-0.948225976075	12.504914493994	2.736995444470
H	-1.903081671009	11.988305543591	2.620574885731
H	-1.057691141625	13.506835646826	2.314898099997
H	-0.199688185519	11.974486462846	2.146961178168
C	-1.643438929351	13.928699768047	5.303651922999
H	-1.516231946577	14.008269124986	6.383997875728
H	-1.465615616176	14.912592039234	4.861712071159
H	-2.684740323262	13.655596084195	5.109583846980
C	-0.614339571048	11.043081005012	5.540178196398
H	-1.467601238353	10.948045772709	6.220341250866

156
(CyNC(H)NCy)4Ti2Cl2 broken symmetry singlet

Ti	-0.330545376978	1.466756736354	-0.173848083244
Ti	0.330535194886	-1.466760352621	0.173804453395
Cl	0.089970362482	0.251181662818	1.946709178569
Cl	-0.089981873714	-0.251186003933	-1.946753625266
N	2.321857842871	-0.691782128427	-0.023752374666
N	1.765310134722	1.576212396704	-0.286623577724
N	-2.321868505837	0.691777768486	0.023720522223
N	-1.765320070195	-1.576216250598	0.286592366316
N	0.742437764059	-3.301549845125	-0.763266590946
N	0.813428558394	-3.073445881768	1.449348079955
N	-0.742441138583	3.301545184570	0.763222434605
N	-0.813439542657	3.073446400690	-1.449392697197
C	2.608091140430	0.572474084603	-0.218367875939
C	1.048639842621	-3.833544742648	0.400196108668
C	-1.048649186568	3.833541887943	-0.400237666746
C	-2.608100589961	-0.572477005323	0.218345672901
H	3.664252588838	0.821793890931	-0.340001201664
H	1.495596219486	-4.831031016860	0.510221837444
H	-1.495600072293	4.831030988395	-0.510259951717

H	-3.664261793924	-0.821794576408	0.339988660178
C	0.981340441768	-3.922515578932	-2.042541931393
C	1.708647685154	-5.258297272919	-1.985275841839
C	-0.324358346963	-4.059126511435	-2.816981228680
H	1.611801180753	-3.231853824378	-2.620348710781
C	1.955746889537	-5.801293933765	-3.386285024821
H	1.103766258241	-5.979830498380	-1.420589228210
H	2.659109436671	-5.150382011532	-1.452241437184
C	-0.089182806910	-4.607508934690	-4.215477566468
H	-0.989169749160	-4.729975223284	-2.258775403406
H	-0.807468109304	-3.079943828716	-2.855379131822
C	0.658408545652	-5.932004932262	-4.171045853535
H	2.466844382553	-6.766634765983	-3.327605335176
H	2.633197973106	-5.122092832548	-3.918258581852
H	-1.042423743965	-4.722682827845	-4.739182449541
H	0.496002835199	-3.880527594592	-4.792014552475
H	0.863024803234	-6.291750058828	-5.183673813859
H	0.023788881422	-6.688275144469	-3.692054970679
C	1.151785452009	-3.503894538582	2.781748389675
C	2.340444640702	-2.715034691425	3.321024009407
C	-0.051171777163	-3.358076453909	3.704886006981
H	1.434881873269	-4.570408748403	2.750246047226
C	2.663819828136	-3.066937529484	4.764805905696
H	2.100475436194	-1.649278438021	3.233614200818
H	3.207327201195	-2.899436475376	2.681371317584
C	0.278004361394	-3.711786440324	5.146679103880
H	-0.392735703526	-2.318550239533	3.641208466247
H	-0.864624015730	-3.985754939767	3.331621798445
C	1.449513146075	-2.890749173273	5.663466155429
H	3.497041602080	-2.452628684659	5.118800800906
H	3.002103781420	-4.109853706755	4.819827928302
H	-0.603972276954	-3.562020804785	5.776281416366
H	0.529633523304	-4.778222265641	5.213733544494
H	1.690864016383	-3.173197788732	6.692506304339
H	1.166187672509	-1.831473691159	5.682779570416
C	-0.981328808744	3.922510785166	2.042499807236
C	0.324383246594	4.059119272347	2.816917959895
C	-1.708634253562	5.258293923496	1.985244082313
H	-1.611782884212	3.231849124147	2.620314485271
C	0.089230928578	4.607502639851	4.215417796478
H	0.989187170523	4.729966592220	2.258701132315
H	0.807492784902	3.079936042104	2.855307602152
C	-1.955709914993	5.801291618330	3.386257042135
H	-1.103760160082	5.979825643394	1.420547674781
H	-2.659104724811	5.150380523732	1.452224761536
C	-0.658358798255	5.932000045313	4.170997375945

H	1.042480374710	4.722674992547	4.739107502525
H	-0.495946900137	3.880522587740	4.791964332148
H	-2.466806192231	6.766633575208	3.327585153132
H	-2.633154085659	5.122092201793	3.918241541331
H	-0.862958415596	6.291745882474	5.183628446546
H	-0.023745196489	6.688268910212	3.691996338856
C	-1.151798131890	3.503901561752	-2.781791640204
C	0.051154804971	3.358087993162	-3.704935260911
C	-2.340461591142	2.715050543574	-3.321069705158
H	-1.434891766394	4.570416252875	-2.750282754708
C	-0.278025126617	3.711806400342	-5.146725453289
H	0.392718480229	2.318561300642	-3.641264638408
H	0.864607813132	3.985764844906	-3.331670908158
C	-2.663839779248	3.066961803062	-4.764848888286
H	-2.100497599686	1.649292691261	-3.233665279302
H	-3.207341194200	2.899454853173	-2.681414541242
C	-1.449536543442	2.890773973173	-5.663513444134
H	0.603949359253	3.562042876537	-5.776331305990
H	-0.529652850389	4.778242974897	-5.213773297561
H	-3.497064743606	2.452657684682	-5.118844549614
H	-3.002120273289	4.109879427863	-4.819864641648
H	-1.690889596559	3.173228073912	-6.692551576370
H	-1.166213654859	1.831497899417	-5.682832461847
C	-2.381570507982	-2.892902917424	0.477125036786
C	-3.162835471408	-3.012741447057	1.782541996146
C	-3.257662956531	-3.317650636570	-0.697319443025
H	-1.556299695529	-3.607350511125	0.535165093507
C	-3.650099524235	-4.440324722814	1.984152165187
H	-4.028573119906	-2.339890186149	1.757889315077
H	-2.534304012433	-2.686185431822	2.612526743093
C	-3.750382631389	-4.745318583743	-0.510829986052
H	-4.119778377081	-2.643408704278	-0.772333500049
H	-2.694669591290	-3.212726001498	-1.625797483352
C	-4.495140772057	-4.908926410960	0.807202337759
H	-4.216580566474	-4.519824861529	2.916668737576
H	-2.781455141902	-5.102960097020	2.090754615817
H	-4.388834894234	-5.039092304753	-1.349235920214
H	-2.887832433752	-5.424162343404	-0.524274622130
H	-4.798985161340	-5.950629641264	0.946827478747
H	-5.418055709248	-4.316337914118	0.772029225784
C	-3.482386976168	1.586994098145	0.056952460056
C	-4.608482392257	1.204030170484	-0.901438838714
C	-4.035749180853	1.767684948715	1.467086669867
H	-3.123535534006	2.563246525209	-0.275228920258
C	-5.690162603496	2.275299185561	-0.897836666423
H	-5.061051045734	0.252987330990	-0.599126834999

H	-4.200135050226	1.054408697313	-1.903257323623
C	-5.132300668546	2.822314228283	1.492919819781
H	-4.436511007105	0.808134021322	1.817885826473
H	-3.220145166872	2.027867746897	2.144493378667
C	-6.242729830645	2.495815264731	0.503949292011
H	-6.494964344543	2.002293281049	-1.586502439216
H	-5.267166914159	3.216624517288	-1.272202881341
H	-5.538038406257	2.924503257700	2.503701621702
H	-4.696445536172	3.795886692336	1.234542862332
H	-6.993170578128	3.291821515208	0.497173838619
H	-6.757457961839	1.583839889174	0.831570784477
C	3.482374729336	-1.587002695955	-0.056959157438
C	4.035810598743	-1.767643852589	-1.467070834901
C	4.608421913257	-1.204091781167	0.901510306234
H	3.123498578092	-2.563264569925	0.275168432048
C	5.132352948972	-2.822283169881	-1.492887317643
H	4.436600805962	-0.808082650989	-1.817809538523
H	3.220240792213	-2.027790522485	-2.144531998131
C	5.690089929193	-2.275373071738	0.897922257976
H	5.061019446977	-0.253041726230	0.599265312147
H	4.200020355335	-1.054510149380	1.903313199018
C	6.242731445474	-2.495836129088	-0.503842731062
H	5.538144755933	-2.924435017898	-2.503651182063
H	4.696474062038	-3.795861849792	-1.234573911398
H	6.494857040715	-2.002405456662	1.586643682578
H	5.267062840648	-3.216709260831	1.272225549712
H	6.993163751826	-3.291850256092	-0.497059285638
H	6.757486710750	-1.583852383676	-0.831398336672
C	2.381562635103	2.892901010584	-0.477134456256
C	3.257559848699	3.317682375513	0.697369254213
C	3.162927957670	3.012717754623	-1.782492934489
H	1.556289898548	3.607340515834	-0.535246696778
C	3.750280876692	4.745350740431	0.510886402380
H	4.119675919985	2.643450931172	0.772467178562
H	2.694493400314	3.212774255699	1.625805739567
C	3.650195022459	4.440300827378	-1.984097008804
H	4.028669435440	2.339874185504	-1.757755484978
H	2.534465811074	2.686135947452	-2.612519178291
C	4.495140215518	4.908936133944	-0.807091560761
H	4.388664955808	5.039149132154	1.349335602921
H	2.887723259382	5.424186622807	0.524248619666
H	4.216748217462	4.519785087536	-2.916571112877
H	2.781553140533	5.102926282047	-2.090782093396
H	4.798986120770	5.950639024468	-0.946715932039
H	5.418057749707	4.316356733654	-0.771833486350

156
(CyNC(H)NCy)4Ti2Cl2 singlet

Ti	-0.305458462299	1.341354042800	-0.158477146165
Ti	0.305458462299	-1.341354042800	0.158477146165
Cl	0.101536930331	0.258898952048	1.950802144947
Cl	-0.101536930331	-0.258898952048	-1.950802144947
N	2.333419873975	-0.674380461250	-0.032882748944
N	1.786551718013	1.582648855318	-0.313652330315
N	-2.333419873975	0.674380461250	0.032882748944
N	-1.786551718013	-1.582648855318	0.313652330315
N	0.684947996558	-3.183762824283	-0.779002080094
N	0.775787998684	-2.969836032823	1.431434078033
N	-0.684947996558	3.183762824283	0.779002080094
N	-0.775787998684	2.969836032823	-1.431434078033
C	2.633232382710	0.584587215461	-0.239812198839
C	0.990036373646	-3.728407116990	0.378206250989
C	-0.990036373646	3.728407116990	-0.378206250989
C	-2.633232382710	-0.584587215461	0.239812198839
H	3.690294492824	0.828393101125	-0.365799289806
H	1.413998129527	-4.737266936951	0.480028712421
H	-1.413998129527	4.737266936951	-0.480028712421
H	-3.690294492824	-0.828393101125	0.365799289806
C	0.922803909781	-3.792005933082	-2.064182712316
C	1.712545108159	-5.092893739063	-2.023892773771
C	-0.384127764991	-3.989502408274	-2.823122812378
H	1.509987510423	-3.069999209313	-2.649298151315
C	1.974993516275	-5.611393774019	-3.431066144879
H	1.146462607150	-5.848006512372	-1.463145142297
H	2.659170223284	-4.943895866679	-1.494418678294
C	-0.136196032594	-4.514447489906	-4.228642672899
H	-1.007901688320	-4.697168640593	-2.263046588658
H	-0.916872631094	-3.036328493869	-2.850548651194
C	0.678217415568	-5.799620412216	-4.205030737935
H	2.533511246123	-6.550838726746	-3.385336918912
H	2.613288562663	-4.894985341970	-3.962667629246
H	-1.087765748866	-4.673557776685	-4.743892470275
H	0.405156430507	-3.753506975892	-4.804221902963
H	0.890187118534	-6.138338647911	-5.223385567804
H	0.087587172506	-6.591815445407	-3.727870345272
C	1.111792285990	-3.422333912256	2.757925892868
C	2.306944648026	-2.651379370833	3.308237172935
C	-0.087513622741	-3.288755256594	3.687183510667
H	1.389344329505	-4.489794169976	2.708234079564
C	2.630660721209	-3.026364870473	4.746172619403
H	2.077009859228	-1.582455524575	3.236283140339
H	3.171333057033	-2.834104788648	2.665452613708

C	0.243968025483	-3.665056959205	5.122859892597
H	-0.429486196313	-2.248659233841	3.640247497445
H	-0.901925799578	-3.910550441727	3.307399908374
C	1.419402172156	-2.856615260711	5.649758674395
H	3.468558360524	-2.422570236739	5.107247146906
H	2.962549263752	-4.072046936169	4.785255714090
H	-0.636376935567	-3.522329970996	5.756437889464
H	0.492288052528	-4.733189423519	5.173145920566
H	1.661110866538	-3.154781573168	6.674280153665
H	1.141125152843	-1.796329200003	5.684830194613
C	-0.922803909781	3.792005933082	2.064182712316
C	0.384127764991	3.989502408274	2.823122812378
C	-1.712545108159	5.092893739063	2.023892773771
H	-1.509987510423	3.069999209313	2.649298151315
C	0.136196032594	4.514447489906	4.228642672899
H	1.007901688320	4.697168640593	2.263046588658
H	0.916872631094	3.036328493869	2.850548651194
C	-1.974993516275	5.611393774019	3.431066144879
H	-1.146462607150	5.848006512372	1.463145142297
H	-2.659170223284	4.943895866679	1.494418678294
C	-0.678217415568	5.799620412216	4.205030737935
H	1.087765748866	4.673557776685	4.743892470275
H	-0.405156430507	3.753506975892	4.804221902963
H	-2.533511246123	6.550838726746	3.385336918912
H	-2.613288562663	4.894985341970	3.962667629246
H	-0.890187118534	6.138338647911	5.223385567804
H	-0.087587172506	6.591815445407	3.727870345272
C	-1.111792285990	3.422333912256	-2.757925892868
C	0.087513622741	3.288755256594	-3.687183510667
C	-2.306944648026	2.651379370833	-3.308237172935
H	-1.389344329505	4.489794169976	-2.708234079564
C	-0.243968025483	3.665056959205	-5.122859892597
H	0.429486196313	2.248659233841	-3.640247497445
H	0.901925799578	3.910550441727	-3.307399908374
C	-2.630660721209	3.026364870473	-4.746172619403
H	-2.077009859228	1.582455524575	-3.236283140339
H	-3.171333057033	2.834104788648	-2.665452613708
C	-1.419402172156	2.856615260711	-5.649758674395
H	0.636376935567	3.522329970996	-5.756437889464
H	-0.492288052528	4.733189423519	-5.173145920566
H	-3.468558360524	2.422570236739	-5.107247146906
H	-2.962549263752	4.072046936169	-4.785255714090
H	-1.661110866538	3.154781573168	-6.674280153665
H	-1.141125152843	1.796329200003	-5.684830194613
C	-2.397345344625	-2.897050864663	0.526687881424
C	-3.188691091827	-2.993602134175	1.828492769824

C	-3.269557741373	-3.346787319016	-0.641504194227
H	-1.570048769094	-3.605970988922	0.603299027850
C	-3.666227042420	-4.420264280031	2.057947894240
H	-4.060595206653	-2.329986037000	1.781436094791
H	-2.571339897879	-2.642778151028	2.656768804935
C	-3.753583991059	-4.774159607901	-0.431299858089
H	-4.135734743703	-2.678956183073	-0.728383354700
H	-2.706871141518	-3.255138708076	-1.571052746685
C	-4.501919443607	-4.919413919634	0.886861234372
H	-4.237006832874	-4.484592528339	2.989037606742
H	-2.793412477134	-5.074192939121	2.182697586739
H	-4.387350537757	-5.087214268537	-1.266316880957
H	-2.886344618229	-5.447130765172	-0.430028582526
H	-4.796960014735	-5.960951420959	1.045422795574
H	-5.429973158436	-4.336025275530	0.836674756906
C	-3.481288755525	1.584321737069	0.042636525412
C	-4.602608429273	1.193963855098	-0.918964803551
C	-4.047501194620	1.792675276833	1.444175152626
H	-3.107882762376	2.550716018486	-0.302050015410
C	-5.678402680037	2.270465041059	-0.943029555687
H	-5.063427817945	0.250966601249	-0.604246999363
H	-4.187197086538	1.024129810985	-1.914548867131
C	-5.142361268750	2.849794557733	1.443033722023
H	-4.453593923249	0.840709729514	1.809194989363
H	-3.237950569742	2.064877148678	2.124051049803
C	-6.244981864087	2.512260851206	0.449378911443
H	-6.476617182598	1.991503882719	-1.636972529427
H	-5.246856142871	3.204650707626	-1.325412400571
H	-5.556043694799	2.968645463076	2.448772274216
H	-4.702950669087	3.818470773279	1.173189385981
H	-6.992498417974	3.310619960251	0.424295992454
H	-6.765814255124	1.606537504755	0.784618659192
C	3.481288755525	-1.584321737069	-0.042636525412
C	4.047501194620	-1.792675276833	-1.444175152626
C	4.602608429273	-1.193963855098	0.918964803551
H	3.107882762376	-2.550716018486	0.302050015410
C	5.142361268750	-2.849794557733	-1.443033722023
H	4.453593923249	-0.840709729514	-1.809194989363
H	3.237950569742	-2.064877148678	-2.124051049803
C	5.678402680037	-2.270465041059	0.943029555687
H	5.063427817945	-0.250966601249	0.604246999363
H	4.187197086538	-1.024129810985	1.914548867131
C	6.244981864087	-2.512260851206	-0.449378911443
H	5.556043694799	-2.968645463076	-2.448772274216
H	4.702950669087	-3.818470773279	-1.173189385981
H	6.476617182598	-1.991503882719	1.636972529427

H	5.246856142871	-3.204650707626	1.325412400571
H	6.992498417974	-3.310619960251	-0.424295992454
H	6.765814255124	-1.606537504755	-0.784618659192
C	2.397345344625	2.897050864663	-0.526687881424
C	3.269557741373	3.346787319016	0.641504194227
C	3.188691091827	2.993602134175	-1.828492769824
H	1.570048769094	3.605970988922	-0.603299027850
C	3.753583991059	4.774159607901	0.431299858089
H	4.135734743703	2.678956183073	0.728383354700
H	2.706871141518	3.255138708076	1.571052746685
C	3.666227042420	4.420264280031	-2.057947894240
H	4.060595206653	2.329986037000	-1.781436094791
H	2.571339897879	2.642778151028	-2.656768804935
C	4.501919443607	4.919413919634	-0.886861234372
H	4.387350537757	5.087214268537	1.266316880957
H	2.886344618229	5.447130765172	0.430028582526
H	4.237006832874	4.484592528339	-2.989037606742
H	2.793412477134	5.074192939121	-2.182697586739
H	4.796960014735	5.960951420959	-1.045422795574
H	5.429973158436	4.336025275530	-0.836674756906
